# Spectral cluster supertree: fast and statistically robust merging of rooted phylogenetic trees

**DOI:** 10.3389/fmolb.2024.1432495

**Published:** 2024-10-30

**Authors:** Robert N. McArthur, Ahad N. Zehmakan, Michael A. Charleston, Yu Lin, Gavin Huttley

**Affiliations:** ^1^ Research School of Biology, The Australian National University, Canberra, ACT, Australia; ^2^ School of Computing, The Australian National University, Canberra, ACT, Australia; ^3^ School of Natural Sciences, University of Tasmania, Hobart, TAS, Australia

**Keywords:** supertree, spectral clustering, rooted phylogenetic trees, phylogenetics, molecular evolution

## Abstract

The algorithms for phylogenetic reconstruction are central to computational molecular evolution. The relentless pace of data acquisition has exposed their poor scalability and the conclusion that the conventional application of these methods is impractical and not justifiable from an energy usage perspective. Furthermore, the drive to improve the statistical performance of phylogenetic methods produces increasingly parameter-rich models of sequence evolution, which worsens the computational performance. Established theoretical and algorithmic results identify supertree methods as critical to divide-and-conquer strategies for improving scalability of phylogenetic reconstruction. Of particular importance is the ability to explicitly accommodate rooted topologies. These can arise from the more biologically plausible non-stationary models of sequence evolution. We make a contribution to addressing this challenge with Spectral Cluster Supertree, a novel supertree method for merging a set of overlapping rooted phylogenetic trees. It offers significant improvements over Min-Cut supertree and previous state-of-the-art methods in terms of both time complexity and overall topological accuracy, particularly for problems of large size. We perform comparisons against Min-Cut supertree and Bad Clade Deletion. Leveraging two tree topology distance metrics, we demonstrate that while Bad Clade Deletion generates more correct clades in its resulting supertree, Spectral Cluster Supertree’s generated tree is generally more topologically close to the true model tree. Over large datasets containing 10,000 taxa and ∼ 500 source trees, where Bad Clade Deletion usually takes ∼ 2 h to run, our method generates a supertree in on average 20 s. Spectral Cluster Supertree is released under an open source license and is available on the python package index as sc-supertree.

## 1 Introduction

The relentless pace of DNA sequence data acquisition has exposed the poor scalability of phylogenetic reconstruction algorithms, establishing that new scalable and accurate algorithms are required for phylogenetic analysis. The well known sensitivity of phylogenetic results to data features that violate models of sequence evolution is increasing the interest in applying non-stationary models to phylogenetic inference (e.g., Dang et al., 2022; Kaehler et al., 2015; Sumner et al., 2012) including estimation of rooted phylogenies (Kaehler, 2017; Yap and Speed, 2005). However, such models increase the number of free parameters and thus worsen computational performance. Theoretical results confirming the statistical consistency of the divide-and-conquer Disk-Covering Method (DCM) (Huson et al., 1999) identified this as a promising candidate for overcoming the robustness versus performance trade-off, illustrated more recently by DACTAL (Nelesen et al., 2012). DCM algorithms fundamentally rely on merging phylogenies estimated from overlapping subsets of the full data. While most prior DCM work has been focused on unrooted trees, rooted topologies can also be handled. Given these considerations, we have sought to resolve one important bottleneck affecting the generalisation of DCM by developing a rooted supertree method that is statistically robust and scalable.

Algorithms for merging phylogenies into a single phylogenetic tree can largely be divided into two categories - supertree methods and consensus tree methods. Consensus tree methods assume the sets of taxa in each of the phylogenies are identical. Supertree methods solve the more general problem where the sets of taxa are not necessarily the same but are overlapping (Steel and Bocker, 2000). A spectral clustering based consensus tree method has been developed for clustering gene trees for multilocus phylogenetic analysis (Yoshida et al., 2019). We will show how spectral clustering can be applied as part of an efficient and statistically robust supertree method.

In this work, we introduce Spectral Cluster Supertree (SCS) for the case of rooted trees. SCS is a method which recursively partitions the set of all taxa in the source trees until a rooted supertree is generated. SCS derives its origins from Min-Cut Supertree (Semple and Steel, 2000), replacing the min-cut step with a substantially more scalable spectral clustering approach (Shi and Malik, 2000) to partition an internal graph. We also extend the method to make use of information such as branch lengths and the depths of taxa in the source trees. These modifications lead to a much more efficient and accurate method, capable of solving problems with hundreds of source trees and ten thousand taxa in the order of seconds.

### 1.1 Related work

Fleischauer and Böcker (2017) performed an extensive systematic comparison among supertree methods in their paper that introduced Bad Clade Deletion (BCD). The comparison evaluated the running time and accuracy of both rooted and unrooted supertree methods. The accuracy over generated datasets was measured by counting the number of splits in the generated supertree that were “true positives” (splits in the generated supertree that should occur), “false positives” (splits in the generated supertree that should not occur) and “false negatives” (splits in the generated supertree that should occur but do not). These values can be used to calculate the well-known $F_{1}$ score.

The methods that were evaluated by the comparison included the Greedy Strict Consensus Merger (GSCM) (Roshan et al., 2003); FastRFS (Vachaspati and Warnow, 2017); Matrix Representation with Parsimony (MRP) (Baum, 1992; Ragan, 1992); SuperFine (Swenson et al., 2012); Combined Analysis using Maximum Likelihood (CA-ML) with RAxML (Stamatakis, 2006); and their own method BCD (Fleischauer and Böcker, 2017). A rooted variant of GSCM exists that generates a supertree only containing clades that are compatible with all of the source trees (Fleischauer and Böcker, 2016). FastRFS (Vachaspati and Warnow, 2017) generates an unrooted supertree optimising the Robinson-Foulds distance (Robinson and Foulds, 1981) to the source trees under a constrained search space using dynamic programming. MRP generates a rooted supertree by performing parsimony analysis on a Baum-Ragan encoding of the source trees (Baum, 1992; Ragan, 1992). Superfine (Swenson et al., 2012) is a meta-method which combines the Strict Consensus Merger (Huson et al., 1999) to merge the source trees together, with another supertree method to resolve polytomies (MRP yielded the best performance); it generates unrooted trees. CA-ML concatenates the sequences used to generate each of the source trees and creates a supertree using maximum likelihood analysis; it has the potential to generate rooted trees depending on the chosen substitution model (e.g., using a strand-symmetric model with IQ-TREE, Minh et al., 2020). The methods were evaluated with dataset sizes varying from 100 to ∼ 7000 taxa.

Of the methods evaluated, CA-ML consistently performed the best in terms of topological accuracy of the constructed trees according to the $F_{1}$ score, generally followed by BCD. However, BCD was much faster than CA-ML. On a particular dataset, BCD usually took under 8 seconds whereas CA-ML took approximately 3 days. On this dataset, all other methods usually took less than a minute to resolve a supertree with the exception of MRP which usually took between 15 min and an hour. BCD was consistently the fastest. The exact ordering of the accuracy of BCD and the other methods depended on the exact dataset, although BCD was most consistently at the top. Of particular importance when dealing with scalability was BCD’s performance on a large dataset containing an average of 5,500 taxa. BCD with an option enabling branch length weighting performed the best in terms of accuracy here. It was also the fastest excluding GSCM (which exhibited very poor topological accuracy), taking 4–8 h. The next fastest non-BCD algorithm was FastRFS, usually taking 8–16 h, but had poor accuracy in comparison. Superfine had the second best non-BCD accuracy and typically took between 16 h and 3 days to resolve a supertree. MRP did not terminate within 14 days and on average exhibited worse accuracy than Superfine.

Spectral Cluster Supertree was created out of the desire for a rooted supertree algorithm that was capable of resolving a supertree containing thousands of taxa in both a fast and topologically accurate manner. Of the rooted supertree algorithms, based on the comparisons by Fleischauer and Böcker (2017), BCD is clearly the most efficient and accurate. *We thus restrict our main comparisons in this paper to be between Spectral Cluster Supertree, and Bad Clade Deletion*.

The BCD (Fleischauer and Böcker, 2017) algorithm seeks to minimise the number of deleted characters in a Baum-Ragan (Baum, 1992; Ragan, 1992) matrix encoding of the source trees so that a consistent supertree is formed. In the matrix, rows represent the taxa, and columns represent the clades, or equivalently internal nodes, of a phylogenetic tree. An entry in the matrix is “1” if the taxon is in the clade, “0” if it is in the tree for the clade but not in the clade, and “?” otherwise. BCD at each iteration aims to delete a locally minimum number of columns from this matrix to yield a supertree.

From the matrix representation, BCD constructs a graph with an edge connecting clade i to taxon j if the corresponding entry in the matrix is 1. Any clades with no 0 entries in their column contain no useful information for the algorithm and are ignored in this process. If the graph is disconnected, the algorithm recurses over the separate components of the graph. The taxa in each of these components belong to different sides of the root in the tree. If the graph is not disconnected, BCD attempts to delete a subset of clades from the graph to make it so. The algorithm partitions the taxa over a minimum-weight cut of a transformation of the graph, only allowing clades to be deleted. The algorithm recurses over the components of the partition until a complete supertree is formed.

BCD also introduces a number of additional strategies to improve the accuracy of the generated supertrees and the time it takes to construct them. This includes a number of weighting strategies for the clades during the min-cut step, which can take into account information including branch lengths and bootstrap values. There is also an optional preprocessing step. A rooted variant of the Greedy Strict Consensus Merger algorithm is used to collect clades that do not contradict with any of the source trees and ensure they are never cut (Fleischauer and Böcker, 2016; Roshan et al., 2003). This step is later referred to as GSCM preprocessing. There is an additional step which reduces the problem size by merging identical clades in the matrix representation. Parallelisation is additionally exploited when finding min-cuts and over the different partitions of the problem.

### 1.2 Using more statistically robust distance measures

It is conventional to use the Robinson-Foulds distance measure (Robinson and Foulds, 1981) to compare tree topologies. In the case of two rooted trees, the measure is determined from the number of clades that do not match between them. The Robinson-Foulds measure is known to exhibit poor statistical behaviour (Böcker et al., 2013; Lin et al., 2011), for instance, moving a single leaf in a caterpillar tree can maximise the metric (Lin et al., 2011). We will later show in the definitions and methods a mapping between the Robinson-Foulds distance, and a rooted variant of the $F_{1}$ score used by Fleischauer and Böcker (2017). Alongside the Robinson-Foulds distance, we also included the Matching Cluster distance (Bogdanowicz and Giaro, 2013). Unlike the Robinson-Foulds distance, the Matching Cluster distance also considers the degree of dissimilarity between the clades that do not match. By capturing more detail in how two topologies differ, it exhibits more robust statistical behaviour.

## 2 Definitions and methods

### 2.1 Preliminaries

A *phylogenetic tree*, T=(V,E) , is a connected, acyclic graph displaying evolutionary relationships over a set of *taxa*, S(T) — the leaves of the graph. Here, V is the set of leaves and internal vertices, and E is the set of edges that connect them. A tree can be *rooted* at an internal vertex to provide an orientation to the tree. In a *rooted bifurcating tree*, every internal vertex of T has degree three except for a special vertex of degree two that is labelled the root. A rooted tree is *multifurcating* if the degree of the root is greater than or equal to two and every other internal vertex of T is greater than or equal to three.

A taxon, t∈S(T) , is said to be a *descendant* of an internal vertex, v∈V , of a rooted phylogenetic tree if the path that connects the root to t passes through v . A *clade*, C , is the set of all descendants of an internal vertex. A clade is called *trivial* if |C|=1 (it is a single leaf) or C=S(T) (the clade for the root of the tree). All other clades are *non-trivial*.

Two taxa u,v∈S(T) are said to belong to a *proper cluster* of T if the unique path connecting u to v does not pass through the root (Semple and Steel, 2000). That is, two taxa belong to a proper cluster if they are both descendants of an identical non-root vertex of T .

For a graph G=(V,E) , a *contraction* of two vertices u,v∈V where (u,v)∈E gives a new graph $G^{\prime }=\left(V^{\prime },E^{\prime }\right)$ formed by removing the edge from E and combining u and v into a single vertex, deleting any parallel edges. For contraction in the context of a weighted graph, all parallel edges except the one of maximal weight are deleted.

We say that a tree, A , is a *subtree* of another tree, B , if A can be obtained from B by deleting all taxa not in A from B and performing a sequence of contractions. If A is a subtree of B , then we say that B *displays* A . A collection of trees is called *compatible* if there exists a phylogenetic tree that displays all of them.

Let *T* be a phylogenetic tree and *X* be a set of taxa. The *induced subtree* $T{|}_{X}$ is the maximally sized subtree of *T* such that $S\left(T{|}_{X}\right)\subseteq X$ . A multiset of trees, $T=\left\{T_{1},T_{2},\ldots ,T_{n}\right\}$ , can be induced on X such that $T{|}_{X}=\left\{T_{1}{|}_{X},T_{2}{|}_{X},\ldots ,T_{n}{|}_{X}\right\}$ . If inducing a subtree would remove all taxa in a tree, it does not appear in the resulting set.

A rooted supertree algorithm is one which takes as its input a multiset of rooted trees T , called *source trees*, and returns a single rooted tree T such that ${⋃}_{i=1}^{n}S\left(T_{i}\right)=S\left(T\right)$ .

### 2.2 Min-Cut supertree

Min-Cut Supertree is a well-studied polynomial-time algorithm for merging rooted phylogenetic trees (Semple and Steel, 2000). It works by recursively partitioning the set of taxa until a rooted tree is formed. There are proven properties of the algorithm, including that if all of the source trees are compatible, then the returned tree displays all of them, and that any triple that is displayed by all of the source trees is displayed in the supertree.

#### 2.2.1 Proper cluster graph

**FIGURE 1:**
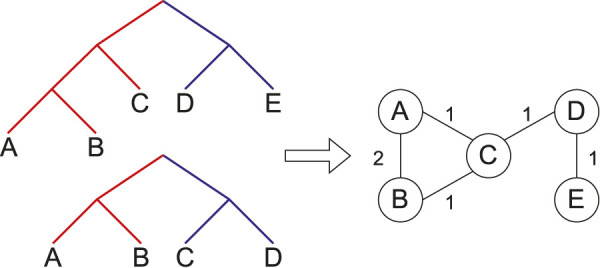
A proper cluster graph (right) for an example set of two source trees (left). Same coloured edges identify taxa belonging to a proper cluster. Here, the trees have unit weights. That is, each edge in the proper cluster graph is weighted by the number of source trees in which the taxa form a proper cluster.

Given a multiset of rooted source trees, $T=\left\{T_{1},T_{2},\ldots ,T_{n}\right\}$ , and an associated weight, $W_{i}$ , for each of the trees (usually set to 1), Min-Cut Supertree constructs the weighted proper cluster graph G=(V,E,w) . The proper cluster graph contains vertices $V={⋃}_{i=1}^{n}S\left(T_{i}\right)$ . An edge, (u,v) , is in E if there exists any tree T∈T for which u and v form a proper cluster. Let I be an indicator function which, given a tree and two taxa, returns one if the taxa form a proper cluster in the tree and zero otherwise. Each edge, (u,v)∈E , is weighted by the sum of the weights of the trees in which u and v form a proper cluster; this is demonstrated by Equation 1. An example proper cluster graph created from two trees is shown in Figure 1.

$$w\left(u,v\right)=\overset{n}{\underset{i=1}{\sum }}W_{i}\cdot I\left(T_{i},u,v\right)$$ (1)

**FIGURE 2:**
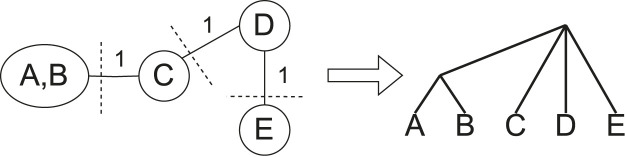
Graphical representation of the partitioning and result merging processes. After contraction, any edge on any min-cut of the proper cluster graph (left) is removed. Min-Cut Supertree is recursively called on the induced subtrees of the components of the proper cluster graph, making the roots of the resulting trees adjacent to a new root vertex (right).

If there are any edges in the proper cluster graph (e∈E) with weight equal to the sum of the weights of the source trees $\left(w\left(e\right)=\sum_{i=1}^{n}W_{i}\right)$ , then every tree supports that proper cluster. Then, when finding a partition of the taxa, taxa in such proper clusters should never be separated. These edges are contracted to ensure this property and to reduce the problem size. Figure 2 (left) shows the proper cluster graph from Figure 1 after such a contraction.

#### 2.2.2 Finding the best partition

If the resulting proper cluster graph is disconnected, then the taxa in each of the components of the graph are always split over the roots of the source trees. Taxa in these components should thus be separated over the root of the supertree. Otherwise, if the proper cluster graph is connected, there is no partition of the taxa for the root of the supertree satisfying all of the source trees. To resolve these cases, every edge that lies on any min-cut of the proper cluster graph is removed. This is analogous to removing groupings of taxa with the weakest support in the source trees so that the graph can be partitioned.

The components of this disconnected graph partition the taxa into n disjoint sets $\left\{X_{1},X_{2},\ldots ,X_{n}\right\}$ . From this partition, n multisets of induced subtrees are generated $\left\{T{|}_{X_{1}},T{|}_{X_{2}},T|X_{n}\right\}$ . Min-Cut Supertree is then recursively called on each of these collections of induced subtrees.

If there are two or fewer taxa remaining when Min-Cut Supertree is called, the method immediately returns the phylogenetic tree containing those taxa. Once the trees generated from the recursive calls are returned, Min-Cut Supertree combines the results by connecting the roots of these trees to a new root vertex. This thus allows Min-Cut supertree to recursively create a complete rooted phylogenetic tree. Figure 2 demonstrates this, continuing the example in Figure 1.

### 2.3 Spectral clustering

Spectral clustering (Ng et al., 2001; Shi and Malik, 2000) is a clustering technique that can be applied to partition a graph over a “bottleneck”. We explain how spectral clustering works from two perspectives.

#### 2.3.1 Random walk perspective

We adapt this point of view from a tutorial on spectral clustering by Von Luxburg (2007). Consider an agent performing a random walk along a graph with n numbered nodes, and without loss of generality assume the graph is connected. The random walk can be represented as a symmetric stochastic matrix P . Let the vector $u=\left(u_{1},u_{2},\ldots ,u_{n}\right)$ represent the initial probability the agent is in each node. The probability the agent is in each node after t time steps is given by $P^{t}u$ . Spectral clustering can be used to partition the graph into separate regions where the agent is most likely to remain trapped in for an extended number of time steps.

As P is a real symmetric matrix, it is eigendecomposable with eigenvalues $\lambda_{i}$ and eigenvectors $v_{i}$ . Sort the eigenvectors by decreasing eigenvalue. By writing u in terms of P ’s eigenvectors (Equation 2), the distribution of the agent after t time steps can be formulated as below.

$$u=\alpha_{1}v_{1}+\alpha_{2}v_{2}+\cdots +\alpha_{n}v_{n}$$ (2)

$$P^{t}u=\lambda_{1}^{t}\alpha_{1}v_{1}+\lambda_{2}^{t}\alpha_{2}v_{2}+\cdots +\lambda_{n}^{t}v_{n}$$ (3)

All eigenvalues of P are less than or equal to 1. The largest eigenvalue is 1, and the associated eigenvector is the stationary distribution π such that Pπ=π . For the symmetric stochastic matrix here, it is $\pi =\left(1/\sqrt{n},\ldots ,1/\sqrt{n}\right)$ . As t→∞ , the distribution of the agent converges to the stationary distribution. The second-largest eigenvalue/eigenvector pair thus represents the slowest part of Equation 3 to decay as it converges to the stationary distribution. The values in this eigenvector corresponds to “bottleneck” regions in the graph in which the agent may remain trapped in for the longest period of time steps. By partitioning this eigenvector into two clusters by, for example, using k -means clustering, the graph is separated over the bottleneck. This process can be applied in a similar fashion, starting from more general types of graphs (Shi and Malik, 2000). It is usually done equivalently over the second-smallest eigenvalue of an alternative representation called the Laplacian matrix; see (Meilă and Shi, 2001; Shi and Malik, 2000; Von Luxburg, 2007) for further reading.

#### 2.3.2 A normalised cut perspective

Another perspective for looking at this problem is from the normalised cut perspective. Let G=(V,E,w) be a weighted graph. Define $W:P\left(V\right)\times P\left(V\right)\to R^{+}$ as the weighting between two sets of vertices given by Equation 4.

$$W\left(A,B\right)=\underset{a\in A,b\in B}{\sum }w\left(a,b\right)$$ (4)

The normalised cut (Shi and Malik, 2000) of G aims to bipartition the vertices into two sets A and B such that the formula given by Equation 5 is minimised.

$$Ncut\left(A,B\right)=\frac{W\left(A,B\right)}{W\left(A,V\right)}+\frac{W\left(A,B\right)}{W\left(B,V\right)}$$ (5)

The solution to this optimisation problem essentially separates the vertices into the two most densely connected regions of the graph across a bottleneck. Minimising this value has been shown to be NP-complete (Shi and Malik, 2000). However, a relaxation of the problem can be solved in polynomial time. The solution to the relaxation is the solution obtained through spectral clustering, yielding an efficient solution relying on eigensolvers (Ng et al., 2001; Von Luxburg, 2007).

### 2.4 Proposed algorithm: spectral cluster supertree

Spectral Cluster Supertree is a supertree method derived from Min-Cut Supertree. Through analysis of the Min-Cut Supertree algorithm, it was determined that the most time-consuming operation was the min-cut step. In particular, the need to remove every edge on any min-cut of the proper cluster graph. The original paper (Semple and Steel, 2000) proposed a test for determining if an edge was in any min-cut of the proper cluster graph. For each edge, if the edge was deleted and the weight of the min-cut of the new graph was equal to the weight of the min-cut of the original graph minus the weight of the edge, then the edge is on a min-cut of the graph. Page (2002) proposed an improvement to this method, instead using Picard and Queryanne’s algorithm (Picard and Queyranne, 1980) to explicitly find all minimum cuts of the graph. However, in practice, finding even a single arbitrary min-cut of the proper cluster graph in this algorithm was too computationally expensive.

**FIGURE 3:**
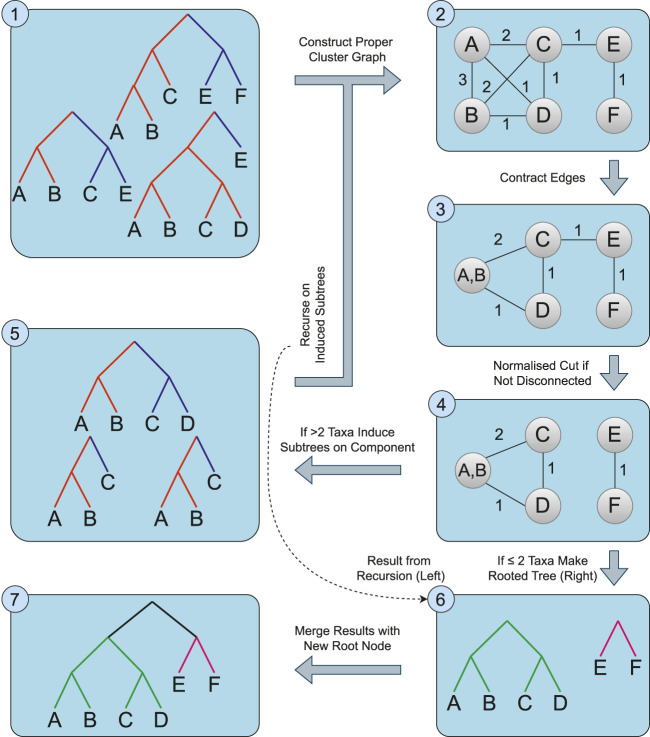
An overview of the Spectral Cluster Supertree Algorithm. The source trees (1) are used to construct the proper cluster graph (2). Edges for proper clusters that only appear together in the source trees are contracted (3). If the graph is not disconnected, spectral clustering is used to take a normalised cut of the graph (4). For any component of the graph with more than two taxa, the source trees are induced on the taxa set and the algorithm recurses on the induced trees (5); otherwise a rooted tree containing the one or two taxa is formed (6). The rooted trees obtained from this step are merged together, connecting the roots of the trees to a new root node (7).

Instead of relying on the (in practice) slow min-cut algorithm to partition the proper cluster graph, we use spectral clustering to efficiently separate the graph into two densely connected components. The remainder of the process is mostly identical to that of Semple and Steel (2000), using these components to induce the new trees for the recursive call. We also include an optimisation from Page’s modified Min-Cut Supertree Page (2002) whereby if only one tree is present on a recursive call, it is returned early and grafted onto the growing tree. We modify the contraction condition for the proper cluster graph to further decrease the problem size. Where Min-Cut Supertree contracted edges representing proper clusters which appeared in every source tree, we instead contract edges representing proper clusters which appear only in every source tree where either of the two taxa are present. An overview of the algorithm is presented in Algorithm 1; Figure 3. We now present additional modifications that allow the method to make use of additional information provided by the source trees.

Algorithm 1 The Spectral Cluster Supertree Algorithm.

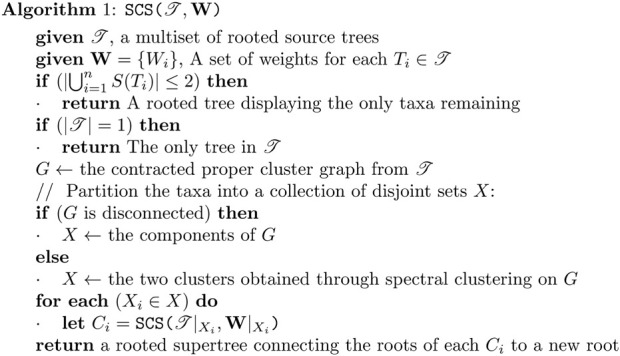

### 2.5 Weighting strategies

BCD (Fleischauer and Böcker, 2017) introduced weighting strategies over the clades of the source trees to enhance the accuracy of their algorithm. Making use of the additional information that could be gleaned from the source trees allowed for more accurate supertree generation. To boost the topological performance of SCS, we outline similar weighting strategies gathered from information of the proper clusters in the source trees to weight the proper cluster graph.

For this part, recall that $T=\left\{T_{1},T_{2},\ldots ,T_{n}\right\}$ is a multiset of rooted source trees, and that S(T) denotes the set of taxa displayed in all of the trees. Like Min-Cut Supertree (Semple and Steel, 2000), we associate each source tree $T_{i}\in T$ with a user-specified weight $W_{i}$ . This can correspond to the user’s confidence in each of the source trees. If none are specified, unit weights are used. Finally, let lca be a function that, given a tree and two taxa, returns the node that is the lowest common ancestor of the two taxa if they are both present in the tree, or the root otherwise. We present two modifications to the weighting function for the proper cluster graph w(u,v) , originally defined by Equation 1.

#### 2.5.1 Depth weighting

SCS works by recursively splitting taxa over a root. The further the lowest common ancestor of a proper cluster is from the root of a source tree, the more likely that proper cluster is to be correct in the true tree. Conversely, the closer the lowest common ancestor of a proper cluster is to the root of a source tree, the weaker the proper cluster is. That is, it is more probable that the two taxa were incorrectly misplaced together on the same side of the root.

Depth weighting can be used when the source trees are provided without branch lengths, or when there is other reason for branch lengths to be ignored. Let d be a function that returns the depth of an internal node in a tree from its root (in terms of the number of edges from the root). When using depth weighting, the weight of each edge in the proper cluster graph for taxa u and v is given by Equation 6.

$$w\left(u,v\right)=\overset{n}{\underset{i=1}{\sum }}W_{i}\cdot d\left(lca\left(T_{i},u,v\right)\right)$$ (6)

#### 2.5.2 Branch length weighting

Branch length weighting works under the same motivation as depth weighting, making use of the branch lengths of the source trees. Let b be a function that returns the branch length distance of an internal node in a given source tree from its root (the root node returns 0). The weight of each edge in the proper cluster graph for taxa u and v is given by Equation 7.

$$w\left(x,y\right)=\overset{n}{\underset{i=1}{\sum }}W_{i}\cdot b\left(lca\left(T_{i},x,y\right)\right)$$ (7)

### 2.6 Implementation

We provide an implementation of our algorithm on GitHub under an open-source license. A Zenodo archive of the repository is available (https://doi.org/10.5281/zenodo.11118433). The algorithm was implemented using Python 3 and is installable as a package, sc-supertree, on PyPI. The Spectral Clustering step of the algorithm was implemented using scikit-learn (Pedregosa et al., 2011). The algorithm can be invoked through the command line using the scs command in its installed environment, taking as input a file name containing line-separated Newick-formatted source trees. The algorithm can also be utilised as a library with the construct_supertree function, taking as input a sequence of cogent3 tree objects (Knight et al., 2007). We also provide a load_trees function to convert a line separated Newick-formatted source tree file into a list of cogent3 trees. Further details on usage, including specifying the weighting strategy, are available in the project’s README.

## 3 Experimental design

We perform extensive comparisons between SCS and BCD to evaluate their statistical and computational performance. Comparisons were made over a number of datasets, both existing and new. Our new dataset differs from those previously studied as they are generated without relying on an outgroup, and in that it aims to mimic what may be encountered during the last stage of divide-and-conquer algorithms such as DACTAL (Nelesen et al., 2012). This also corresponds to the practical application of inferring the tree root without prior knowledge of the phylogeny. An extended description of the datasets is presented below and summarised in Table 1.

**TABLE 1:** A summary of key properties of the datasets used to evaluate the supertree methods.

Dataset	Summary
SMIDGenOG	Aims to emulate data collection processes used by systematists. Each problem contains many densely sampled clade-based source trees and a single widely sampled scaffold tree containing “Scaffold Factor” percent of the taxa
SMIDGenOG-5500	Similar to SMIDGenOG but at a much larger scale with each problem containing on average 5,500 taxa. Unlike SMIDGenOG, each problem contains 5 widely sampled scaffold trees with 100 randomly selected taxa each
SuperTriplets	Explores the effect of the number source trees, and percentage of taxa missing from each of the source trees on supertree reconstruction
SCS-Exact	Evaluates supertree reconstruction under conditions that may be encountered by divide-and-conquer methods. Simply applies REC-I-DCM3 to decompose a known model tree into overlapping source trees. As there are no conflicts, all supertree methods should be able to quickly construct the correct supertree
SCS-DCM-IQ	Evaluates supertree reconstruction under conditions which may be encountered at the final step of divide-and-conquer methods prior to convergence. For the taxa sets generated by applying REC-I-DCM3 to the model tree, uses IQ-TREE 2 to form a collection of overlapping maximum likelihood source trees

### 3.1 Datasets

#### 3.1.1 SMIDGenOG

The SMIDGenOG dataset was generated by Fleischauer and Böcker (2016) using the SMIDGen protocol (Swenson et al., 2010) in a rooted context using outgroup rooting. The SMIDGen protocol emulates data collection processes typically used by systematists, having many densely-sampled clade-based source trees, and a more widely sampled scaffold tree representing relations at a higher taxonomic level. Each model tree was paired with a single such *scaffold tree* and a number of densely sampled *clade-based source trees*. The scaffold tree samples a percentage of taxa, the scaffold-factor, uniformly at random over the entire tree. Five “universal” genes (which originate at the root and do not become extinct) were simulated to estimate maximum-likelihood scaffold trees for the dataset. For the clade-based trees, a gene birth-death process (Swenson et al., 2010) was used to select 200 subtrees to simulate non-universal genes. All genes were simulated under a GTR + Gamma + Invariant sites process. Alignments for each of the clade-based trees were created by selecting a clade of interest (under a birth node process according to the SMIDGen protocol), and concatenating three non-universal gene sequences with the highest taxa coverage. An outgroup with all three non-universal genes present was also added to the alignment. Maximum likelihood trees were estimated over the alignments with RAxML (Stamatakis, 2006). All source trees were rooted with the outgroup, and the outgroup was subsequently removed. There are 30 model trees with corresponding source trees for every combination of 100, 500, and 1,000 taxa with a scaffold factor of 20%, 50%, 75% and 100%.

#### 3.1.2 SMIDGenOG-5500

The SMIDGenOG-5500 dataset was generated by Fleischauer and Böcker (2017) as a large-scale variant of the SMIDGenOG dataset. It was created using a similar methodology to the SMIDGenOG dataset with 10 model trees with an average of 5,500 taxa. Each model tree was paired with 500 densely sampled clade-based source trees with 75–125 taxa. Due to the size of the dataset (to ensure the maximum likelihood process could run in a reasonable time frame), each model tree was also paired with five sparsely-sampled scaffold source trees with 100 randomly selected taxa. This differs to SMIDGenOG where a single scaffold tree with varied scaffold factors were used (Fleischauer and Böcker, 2016).

#### 3.1.3 SuperTriplets

The SuperTriplets dataset was generated by Ranwez et al. (2010) to explore the effect of both the size and number of source trees on supertree construction. It contains 100 model trees with 101 taxa each (including an outgroup). The source trees are divided into a number of deletion rates d∈{25%,50%,75%} and a constant k∈{10,20,30,40,50} . In the data generation process, each model tree was duplicated 50 times with varied branch lengths. Sequence alignments were simulated on these duplicated trees. Maximum likelihood trees were estimated from these 50 alignments, each under the constraint of d percent of the ingroup taxa being removed. The first k of these trees formed the set of source trees.

#### 3.1.4 SCS datasets

We generated the SCS datasets to evaluate supertrees in a setting as may be encountered by rooted variants of divide-and-conquer methods for phylogenetic reconstruction (e.g., Nelesen et al., 2012). In this case, the data generation process does not necessarily rely on an outgroup.

Ten model trees (with 500, 1,000, 2000, 5,000 and 10,000 taxa) were generated following a birth-death process with a birth rate of 1.0 and a death rate of 0.2. The rooted ultrametric trees were initially scaled such that every tip was of distance one from the root. Similar to the SMIDGen protocol (Swenson et al., 2010), branch lengths were then scaled by a random scaling factor. At the root, the scaling factor was 1.0 and the scaling factor evolved down different parts of the tree by adding a number from a normal distribution with a mean of 0 and a standard deviation of 0.05. The scaling factor was additionally bounded in this process between 0.05 and 8.0 to guard against excessively long or short branch lengths following the SMIDGen protocol.

For each of the model trees, cogent3 (Huttley, 2020) was used to simulate a sequence alignment of length 10,000 under a strand-symmetric general nucleotide Markov substitution process under which the root is identifiable (Kaehler, 2017). The alignment length was chosen to allow for a sufficient level of accuracy when estimating the source trees. The parameters for the process were estimated from a sequence alignment of three bacterial species (Kaehler et al., 2015).

Rec-I-DCM3 (Roshan et al., 2004) is a method that can be used to decompose a tree into overlapping subsets of taxa. We have made an implementation of this algorithm in Python publicly available (https://doi.org/10.5281/zenodo.11118313). We used Rec-I-DCM3 to partition each model tree into overlapping subsets of taxa of maximal sizes 50 and 100. We extracted the subtrees for these partitions from the model tree to form a collection of source trees. This forms a simple dataset we call the “SCS-Exact” dataset, from which all methods should always be able to reconstruct the true tree.

For the more interesting second dataset, for each subset in the partition of taxa formed by Rec-I-DCM3, the corresponding simulated sequences were extracted. IQ-TREE 2 (Minh et al., 2020), under a strand symmetric model, was used to fit a rooted tree to these sequences. The set of trees generated for a partition formed the source trees for the “SCS-DCM-IQ” dataset.

### 3.2 Distance measures

We measure the topological accuracy of supertrees generated by the evaluated methods using the standard Robinson-Foulds distance (Robinson and Foulds, 1981), but also the more statistically robust Matching Cluster distance (Bogdanowicz and Giaro, 2013). For this paper, we use a definition of Robinson-Foulds that is the cardinality of the set containing the symmetric difference of the clades between the two compared trees. Let C be a function mapping trees to their set of clades, and ⊕ denote the symmetric difference operator. The Robinson-Foulds distance is given by Equation 8.

$$RF\left(T_{1},T_{2}\right)=|C\left(T_{1}\right)⊕C\left(T_{2}\right)|$$ (8)

**FIGURE 4:**
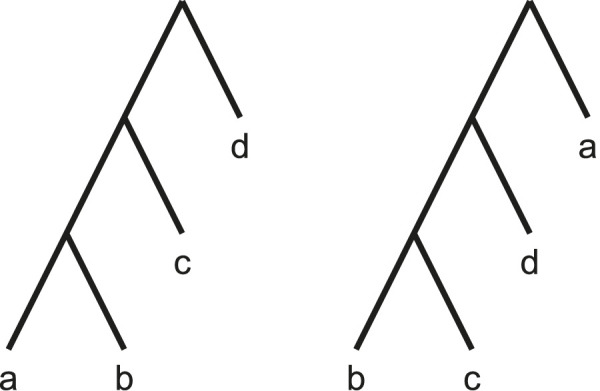
By moving a single leaf (a) to the root of the tree, the Robinson-Foulds distance is maximised.

The Robinson-Foulds distance, while quick to compute, is known to exhibit poor statistical behaviours (Lin et al., 2011; Böcker et al., 2013). For instance, it is known to saturate quickly. This means similar topologies differing by only a single leaf can maximise this metric, as illustrated by Figure 4.

Consider the $F_{1}$ score used by Fleischauer and Böcker (2017), and adapt it to the rooted case (the unrooted version and unrooted Robinson-Foulds follows similarly). This adaptation can be done by letting “true positives” ( TP ) refer to clades in both the supertree and model tree; “false positives” ( FP ) refer to clades in the supertree but not the model tree; and “false negatives” ( FN ) refer to clades in the model tree but not the supertree. The $F_{1}$ score is given by Equation 9 (we include an example calculation in the Supplementary Material). The Robinson-Foulds distance measure can then simply be calculated as RF=FP+FN . Further, there is a direct mapping between the Robinson-Foulds distance and $F_{1}$ score scaled by the number of correctly identified clades as given by Equation 10.

$$F_{1}=\frac{2TP}{2TP+FP+FN}$$ (9)

$$RF=2TP\left(\frac{1}{F_{1}}-1\right)$$ (10)

Further, the number of non-root internal vertices in the supertree is equal to the sum of the number of true positive and false positive clades. In the case of a fully resolved tree with n taxa containing no polytomies, there are n−2 such internal vertices and $FP=FN=\frac{RF}{2}$ . Thus, $TP=n-2-\frac{RF}{2}$ and, treating the number of taxa as constant, there is an exact linear mapping between the $F_{1}$ score and Robinson-Foulds distance given by Equation 11. This implies that under this scenario, the $F_{1}$ score and Robinson-Foulds distance are, in effect, measuring the same relation. Though this exact mapping assumes no polytomies, in practice when we calculated the Robinson-Foulds distance and $F_{1}$ score they told very similar stories in the results. As such, we only report the Robinson-Foulds distance of these two in our comparisons and include the $F_{1}$ score in the Supplementary Material.

$$RF=2\left(n-2\right)\left(1-F_{1}\right)$$ (11)

**FIGURE 5:**
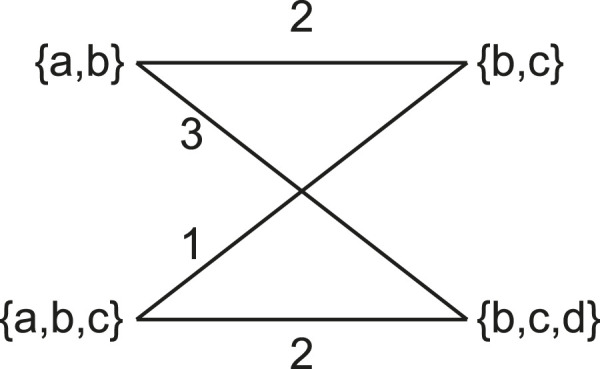
The Matching Cluster distance is calculated by solving the min-weight matching problem of a bipartite graph. Each side of the bipartite graph contains the clusters of the respective trees. Edges are weighted by the cardinality of the symmetric distance of the sets it connects.

We also include the more statistically robust Matching Cluster distance in our comparisons. The Matching Cluster distance (Bogdanowicz and Giaro, 2013) is similar to the β distance (Boorman and Olivier, 1973). Rather than considering only exact matches of clades, it also takes into consideration the degree of dissimilarity between the clades. It does this by solving a min-weight matching problem between the symmetric differences of each pair of clades in the two trees. For the example in Figure 4, the non-trivial clusters are {{a,b},{a,b,c}} and {{b,c},{b,c,d}} respectively. Figure 5 shows a bipartite graph between the non-trivial clusters of the two trees. The edges are weighted by the cardinality of the symmetric difference of the clusters. The solution to the min-weight matching problem here is 4. The maximum possible Matching Cluster distance for bifurcating trees with four taxa here is 6.

When considering multifurcating trees, one tree may have fewer clades than the other. This could mean one side of the graph in the min-weight matching problem could have fewer vertices than the other. The Matching Cluster distance generalises to such scenarios by adding empty sets to the side with fewer vertices until the number of vertices on each side are equal.

The Matching Cluster distance has been shown as more statistically robust than the Robinson-Foulds distance for general tree topologies (Bogdanowicz and Giaro, 2013). That said, for bifurcating trees, the Robinson-Foulds distance and Matching Cluster distance are equal if the weights of the edges for clusters are replaced by 0 if they do match, and two if they do not. See Smith (2020) for further reading on distance measures.

### 3.3 Experiments

Experiments were performed using the Australian NCI’s Gadi (https://nci.org.au), running on a single core of an Intel(R) Xeon(R) Platinum 8268 CPU @ 2.90 GHz with 16 GB of RAM. SCS, BCD with and without the Greedy Strict Consensus Merger (GSCM) preprocessing, and Min-Cut Supertree (MCS) were evaluated over all of the datasets. We used our own Python implementation for SCS and MCS, and the Java implementation of BCD by Fleischauer and Böcker (2017). A modified version of Min-Cut Supertree was used that included our weighting strategies for the proper cluster graph. We also allowed MCS to find only a single arbitrary min-cut of the proper cluster graph, rather than all min-cuts, so the problems could be solved within a reasonable time. Branch length weighting was utilised across all methods where possible (except for the SuperTriplets dataset). Otherwise, depth weighting for our method, or unit weighting for BCD, was used. The CPU time to resolve each supertree was recorded, as well as the Robinson-Foulds Distance and Matching Cluster Distance when compared to the model tree ( $F_{1}$ score results are included in the Supplementary Material). If a method did not complete a dataset under specific parameters within a wall time of 48 h, it was terminated early. We have made the code used to process and evaluate the experiments publicly available (https://doi.org/10.5281/zenodo.11118313). It also includes a script for downloading the datasets.

## 4 Results

### 4.1 Spectral cluster supertree vs. Min-Cut supertree

**FIGURE 6:**
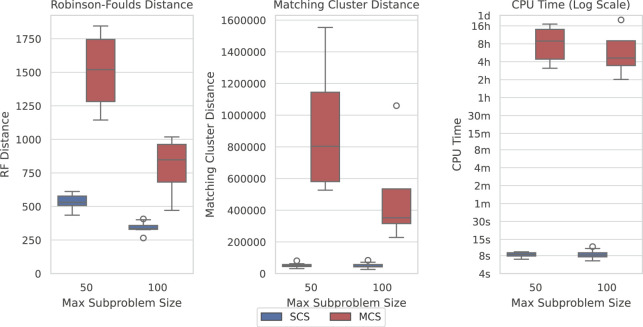
SCS outperforms MCS on the SCS-DCM-IQ dataset with 5,000 taxa. Lower is better for all graphs. Time results are shown on a log scale. Each parameterisation of the dataset contains 10 problems. While SCS solved all problems, MCS only solved 4/10 within the time limit for each of the maximum subproblem sizes.

Here, we show how SCS is a significant improvement over Min-Cut Supertree across all measures. Figure 6 displays the difference in the recorded measures between the two methods over the largest dataset where Min-Cut Supertree resolved at least some problems (4/10 for each of the maximum subproblem sizes). Where SCS takes a matter of seconds to solve a problem, Min-Cut Supertree can take from 2 hours to close to a full day. SCS additionally outperforms Min-Cut Supertree in every metric—consistently producing more correct clades in the generated supertree (from the Robinson-Foulds Distance), and markedly superior general topological accuracy from the Matching Cluster Distance.

The results shown in Figure 6 are similar across most datasets, though not necessarily to the same extent with regard to time on the smaller datasets. MCS only outperformed SCS with respect to time on the SCS-Exact dataset, where importantly no min-cut or spectral clustering calls are required, and all methods recovered the true tree. On the largest SCS-Exact problems, both methods took around 6–10 s. This illustrates the extent at which the min-cut component of the MCS impacts its computational efficiency. SCS always dominated MCS with respect to topological accuracy under both distance metrics on all other datasets. Going forward, we thus only compare the performance of SCS to BCD.

### 4.2 Spectral cluster supertree vs. Bad Clade Deletion

All methods could construct the correct supertree in the SCS-Exact dataset, though GSCM preprocessing made BCD sometimes take hours for the largest problems. Without GSCM, BCD finished in a few seconds. Note that this is contrary to the time results for the other datasets shown in the figures. We now show the results for SCS compared with BCD with and without GSCM preprocessing over each of the datasets.

**FIGURE 7:**
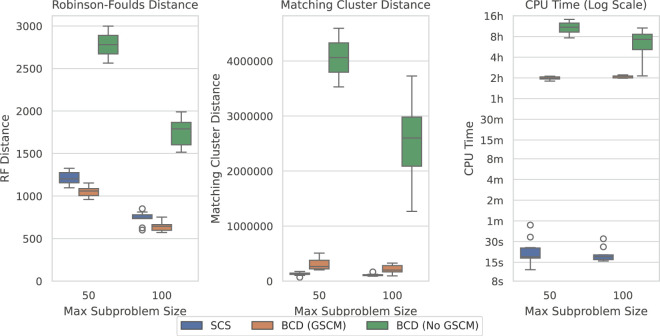
SCS vs. BCD on the SCS-DCM-IQ dataset with 10,000 taxa. The dataset emulates what may be encountered when using divide-and-conquer algorithms for phylogenetic reconstruction. Each parameterisation of the dataset contains 10 problems. BCD without GSCM processing only solved the first two and six problems within the timeout for the 50 and 100 max subproblem sizes respectively. The other methods solved all ten problems.

Figure 7 shows the results over the SCS-DCM-IQ datasets with 10,000 taxa. Results for the other taxa counts follow the same pattern (see Supplementary Figures S2–S5). The figure shows that with GSCM preprocessing, BCD outperforms SCS in terms of the Robinson-Foulds distance. This metric measures the number of clades that are different (no matter how similar) between the model tree and generated supertree. SCS however outperforms BCD under the Matching Cluster distance, which measures more generally how similar the overall topologies of the tree are—taking into account the degree of similarity/dissimilarity in the clades. SCS is also vastly superior here compared to BCD in terms of the time taken to solve the problems. Where BCD takes multiple hours per problem instance, SCS can solve these problems in less than a minute—usually in under 20 seconds.

**FIGURE 8:**
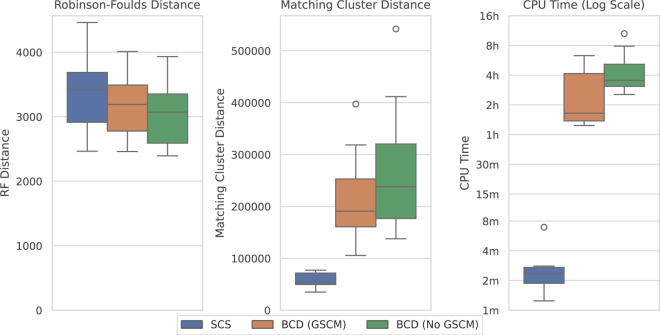
SCS vs. BCD on the SMIDGenOG-5500 dataset. The dataset applies the SMIDGen protocol to mimic what may be encountered by systematists at a large scale. As there is no parameterisation for this dataset, no *x*-axis is displayed. All methods solved all ten problem instances within the timeout.

Figure 8 compares the results of SCS to BCD under the large SMIDGenOG-5500 dataset. The results follow a similar pattern to the SCS-DCM-IQ dataset. BCD still outperforms SCS in terms of the Robinson-Foulds Distance, though the difference is less pronounced. SCS again outperforms BCD in terms of the Matching Cluster distance, which compares the tree topologies more generally. SCS is still a vast improvement over BCD in terms of the time required to solve the problems, taking minutes, where the others can take multiple hours.

**FIGURE 9:**
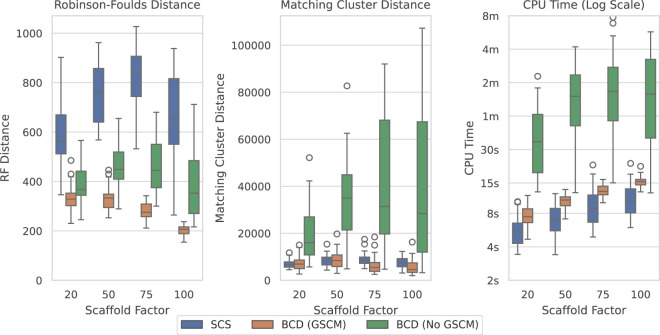
SCS vs. BCD on the SMIDGenOG dataset with 1,000 taxa. The dataset aims to imitate data curation processes of systematists. The SMIDGenOG dataset contains one scaffold tree sampling over “Scaffold Factor” percent of the taxa, as well as many densely sampled clade-based source trees. Each parameterisation of the dataset contains 30 problems.

Figure 9 shows the results over the largest original SMIDGenOG dataset with 1,000 taxa and different scaffold factors (the percentage of taxa sampled for the scaffold trees). This dataset shows the best results for BCD when compared to SCS. BCD outperforms SCS in terms of the Robinson-Foulds Distance. The results are roughly even in terms of Matching Cluster Distance for the smaller two scaffold factors, though BCD with GSCM preprocessing is slightly ahead with the higher two scaffold factors. SCS is slightly ahead with respect to time compared with BCD with GSCM preprocessing. However, problems here are being solved in the order of seconds and the difference is not significant for practical purposes. Results are similar for the 100 and 500 taxa counts (see Supplementary Figures S6, S7), with BCD being faster on the 100 taxa dataset.

**FIGURE 10:**
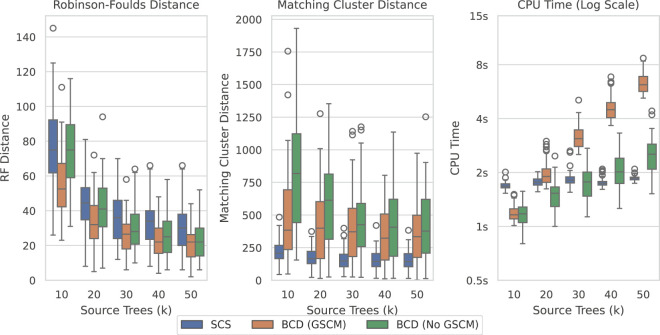
SCS vs. BCD on the SuperTriplets dataset with a deletion rate of 50%. The SuperTriplets dataset explores the effect of percentage of taxa in the source trees, and number of source trees, on supertree construction. Each parameterisation of the dataset contains 100 problems. Results are variable depending on the deletion rate, and results for the others are included in the appendix.

Figure 10 shows the results over the SuperTriplets dataset with a 50% deletion rate (percentage of taxa removed when generating each source tree). The SuperTriplets dataset shows a significant amount of variance over the different deletion rates. With a 50% deletion rate, SCS generally performs worse with respect to the Robinson-Foulds Distance, but better in terms of Matching Cluster distance. With a 75% deletion rate (Supplementary Figure S9), SCS generally outperforms BCD in all distance metrics. For the 25% deletion rate (Supplementary Figure S8), BCD outperforms SCS in terms of the Robinson-Foulds distance. The central distribution of the Matching Cluster distance is roughly identical, with BCD achieving better minimum values, though SCS achieves better maximum values. The time results here are all low enough to not make much of a difference for practical purposes.

Full figures for the other parameterisations of the datasets are available in the appendix. The raw results have been archived on Zenodo (https://doi.org/10.5281/zenodo.11118313).

## 5 Discussion

The Spectral Cluster Supertree (SCS) algorithm for merging rooted phylogenetic trees exhibits comparable or markedly better statistical and computational performance than current approaches. While Min-Cut Supertree (MCS) was demonstrated impractical for modern sized data sets, Bad Clade Deletion (BCD) was far more efficient when used in conjunction with its GSCM pre-processing step. Under most conditions examined, particularly for large problem size, SCS was orders of magnitude faster than BCD. Comparison of the statistical performance of the algorithms was sensitive to the topology distance metric used. Thus, judgement of the statistical merits of SCS relative to competing approaches hinges on the properties of the Robinson-Foulds and Matching Cluster distance metrics.

### 5.1 SCS statistically outperforms BCD on most datasets

In terms of Robinson-Foulds distance, BCD with GSCM preprocessing almost always outperformed SCS or were otherwise roughly equivalent. However, the Robinson-Foulds distance metric suffers from poor statistical qualities. It can saturate quickly; it is possible for trees that are identical minus the placement of a single leaf to maximise this distance metric. It simply compares how many of the clades are an exact match between the model tree and supertree, without considering the degree of similarity or dissimilarity between the clades.

The Matching Cluster distance is a more statistically robust distance measure when comparing tree topologies. It gathers the clades of the model tree and supertree, and measures the degree of dissimilarity between the clades of these two sets (based on the size symmetric difference of the clades, or in other words, how many taxa in the clades are different). Having found the optimal pairing of clades between these sets, the distance measure is calculated. By considering the degree of dissimilarity between the clades of the trees, rather than whether they match or not (as per Robinson-Foulds), it is more robust. If instead the degree of dissimilarity was measured as two if the clades do not match and 0 otherwise, the distance measure would become identical to Robinson-Foulds.

SCS frequently outperformed BCD with respect to the Matching Cluster distance on all datasets except the SMIDGenOG dataset, and SuperTriplets dataset with a deletion rate of 25% . The median improvement of the Matching Cluster distance over each parameterisation of the datasets was measured on a problem by problem basis. By this measure, SCS performed 1.10–2.58 times better than BCD on the SCS-DCM-IQ dataset; 3.45 times better than BCD on the SMIDGenOG-5500 dataset; and from 1.93–2.25 to 2.60–6.60 times better on the SuperTriplets dataset with a deletion rate of 50% and 75% respectively. SCS and BCD performed similarly on the SMIDGenOG dataset with 1,000 taxa and scaffold factors of 20% and 50% with the median improvement of SCS over BCD being 1.03 and 1.00 times respectively. On all other parameterisations of the SMIDGenOG dataset, the median improvement of BCD over SCS was 1.07–1.68 times. BCD also performed 1.18–1.43 times better on the SuperTriplets dataset with a deletion rate of 25% .

It is somewhat curious, on first consideration, the discrepancy between the SMIDGenOG and SMIDGenOG-5500 results given the underlying data was simulated under a similar protocol. The primary difference between the two datasets here is the number of taxa in the scaffold tree. Due to practical limitations, SMIDGenOG-5500 contains five scaffold trees with 100 taxa each (less than 2% of the taxa each on average) and SMIDGenOG contains a single scaffold tree containing either 20% , 50% , 75% or 100% of the taxa. It also appears the rate at which BCD improves on SCS decreases as the scaffold factor decreases. Further, the effect of the deletion rate under the Supertriplets dataset indicates that the more taxa that are removed from each of the source trees (higher deletion rate), the more topologically accurate SCS is compared to BCD.

The algorithmic properties of the two methods could potentially explain this relationship. BCD effectively aims to delete a minimum number of clades from the source trees when conflicts arise to construct a supertree. Importantly, every clade in the supertree must also be a clade in one of the source trees (which leads to lower in comparison Robinson-Foulds distances). Having a strong backbone with a widely sampled scaffold tree can support this. When conflicts arise in SCS, however, we consider the degree to which the source trees support taxa appearing on the same side of a prospective root. We partition the taxa, into two groups with maximal support within each group but minimal support between the two groups. This is clearly quite effective in practice, but it may not count as heavily, in comparison, the presence of a strong backbone. On further examination. we found that by increasing the tree weight associated with the scaffold tree in the SMIDGenOG dataset, the accuracy of the SCS method improved significantly (sometimes better than BCD). The improvement was particularly strong for high scaffold factors. This supports our hypothesis for the explanation of the relationship here. However, as BCD also supports tree weights and to avoid overfitting these extra parameters to the reported accuracy, we do not include these results in our comparisons.

When considering practical applications of the algorithms on large datasets, the choice of method is clear. For large phylogenetic reconstruction problems, the time required to perform a full maximum likelihood analysis over sequences of interest is far too computationally taxing. In these scenarios (as in the SMIDGenOG-5500 dataset), the presence of a large scaffold tree can potentially be infeasible. Divide-and-conquer methods relying on the Disk-Covering Method (Roshan et al., 2004), breaking down the problem into small overlapping subproblems, may thus be required to make computation possible. In these situations, it is clear SCS performs best with respect to topological accuracy, though BCD may still be useful for smaller problems with a strong scaffold tree.

### 5.2 SCS displays superior computational efficiency over BCD in serial

SCS is also vastly more efficient in terms of CPU Time compared to BCD. For the largest problems in the SCS-DCM-IQ dataset, SCS could solve problems (that took BCD roughly 2 h) in under a minute—usually in less than 20 s (median speedup of 386 and 409 times for maximum subproblem sizes of 50 and 100 respectively). For the SMIDGenOG-5500 dataset, where BCD took 1–8 h to solve each problem, SCS took only 1–8 min (median 58 times speedup). Ignoring problems which took both BCD (GSCM) and SCS under 15 seconds to solve, the median speedup over BCD under each combination of parameters of all the datasets ranged from 12 to 409 — the greatest speedups on the largest datasets. The only problems BCD beat SCS on speed, SCS still solved in only 1–2 s. SCS is clearly the superior choice in terms of time needed to fully resolve a supertree.

It must be noted that the results illustrated in the figures were obtained from running the experiments on a single CPU core. BCD has support for multi-threading, which is performed over both min-cut computations and recursive calls. In the current version of SCS, concurrency is only utilised within NumPy operations (Harris et al., 2020) and during a part of the spectral clustering step as implemented in scikit-learn (Pedregosa et al., 2011). There is room for further parallelisation in SCS, however. Similar to how BCD parallelises across independent recursive calls, the same can be done trivially for SCS. This parallelisation was trialled, but the improvement was minor on these problem sizes due to the associated overhead, and the time domination of performing the first and largest split. Parallelisation will likely have a much more beneficial effect for even larger problems than we have tested, and we accordingly leave a full comparison of the parallel versions for future work. Due to BCD’s parallelisation over solving min-cuts in addition to the subproblems, greater speedup may be obtained, particularly on these problem sizes. However, SCS improves on the time results of BCD over large problems sizes to such a significant extent that this difference may not matter. This is especially true when considering limitations such as Amdahl’s law (which gives a theoretical asymptotic limit to the speedup of a program as the number of processors are increased).

## 6 Conclusion

We presented a new algorithm, Spectral Cluster Supertree, for merging overlapping rooted phylogenetic trees. Our algorithm is significantly faster than Bad Clade Deletion on large problem sizes, taking on average 20 s where Bad Clade Deletion took ∼ 2 h on one dataset, and 1–8 min where Bad Clade Deletion took 1–8 h on another. While Bad Clade Deletion can sometimes display a superior topological accuracy on datasets containing large scaffold trees, on most datasets Spectral Cluster Supertree is more accurate. Of particular importance, Spectral Cluster Supertree was more topologically accurate than Bad Clade Deletion on large problems, or otherwise those where the number of taxa in each of the source trees may be low in proportion to the total number of taxa. This may be especially valuable for large problems where divide-and-conquer methods for phylogenetic reconstruction could be necessary for computational feasibility. We leave further comparisons with respect to parallel implementations for future work, where larger datasets than those currently considered are necessary for proper investigation.

## Data Availability

The datasets presented in this study can be found in online repositories. The names of the repository/repositories and accession number(s) can be found below: https://doi.org/10.5281/zenodo.11118022.

## References

[B1] Baum B. R. (1992). Combining trees as a way of combining data sets for phylogenetic inference, and the desirability of combining gene trees. Taxon 41, 3–10. 10.2307/1222480

[B2] Böcker S. Canzar S. Klau G. W. (2013). “The generalized robinson-foulds metric,” in Algorithms in bioinformatics: 13th international workshop, WABI 2013, sophia antipolis, France, september 2-4, 2013. Proceedings 13 (Springer), 156–169.

[B3] Bogdanowicz D. Giaro K. (2013). On a matching distance between rooted phylogenetic trees. Int. J. Appl. Math. Comput. Sci. 23, 669–684. 10.2478/amcs-2013-0050

[B4] Boorman S. A. Olivier D. C. (1973). Metrics on spaces of finite trees. J. Math. Psychol. 10, 26–59. 10.1016/0022-2496(73)90003-5

[B5] Dang C. C. Minh B. Q. McShea H. Masel J. James J. E. Vinh L. S. (2022). nQMaker: estimating time nonreversible amino acid substitution models. Syst. Biol. 71, 1110–1123. 10.1093/sysbio/syac007 35139203 PMC9366462

[B6] Fleischauer M. Böcker S. (2016). Collecting reliable clades using the greedy strict consensus merger. PeerJ 4, e2172. 10.7717/peerj.2172 27375971 PMC4928488

[B7] Fleischauer M. Böcker S. (2017). Bad clade deletion supertrees: a fast and accurate supertree algorithm. Mol. Biol. Evol. 34, 2408–2421. 10.1093/molbev/msx191 28873954 PMC5850620

[B8] Harris C. R. Millman K. J. van der Walt S. J. Gommers R. Virtanen P. Cournapeau D. (2020). Array programming with NumPy. Nature 585, 357–362. 10.1038/s41586-020-2649-2 32939066 PMC7759461

[B9] Huson D. H. Nettles S. M. Warnow T. J. (1999). Disk-covering, a fast-converging method for phylogenetic tree reconstruction. J. Comput. Biol. 6, 369–386. 10.1089/106652799318337 10582573

[B10] Huttley G. A. (2020). Cogent3: comparative genomics toolkit. 10.5281/zenodo.4542532

[B11] Kaehler B. D. (2017). Full reconstruction of non-stationary strand-symmetric models on rooted phylogenies. J. Theor. Biol. 420, 144–151. 10.1016/j.jtbi.2017.03.007 28286217

[B12] Kaehler B. D. Yap V. B. Zhang R. Huttley G. A. (2015). Genetic distance for a general non-stationary markov substitution process. Syst. Biol. 64, 281–293. 10.1093/sysbio/syu106 25503772 PMC4380038

[B13] Knight R. Maxwell P. Birmingham A. Carnes J. Caporaso J. G. Easton B. C. (2007). Pycogent: a toolkit for making sense from sequence. Genome Biol. 8, 1–16. 10.1186/gb-2007-8-8-r171 PMC2375001 17708774

[B14] Lin Y. Rajan V. Moret B. M. (2011). A metric for phylogenetic trees based on matching. IEEE/ACM Trans. Comput. Biol. Bioinforma. 9, 1014–1022. 10.1109/TCBB.2011.157 22184263

[B15] Meilă M. Shi J. (2001). “A random walks view of spectral segmentation,” in International workshop on artificial intelligence and statistics (Key West, Florida, USA: PMLR), 203–208.

[B16] Minh B. Q. Schmidt H. A. Chernomor O. Schrempf D. Woodhams M. D. Von Haeseler A. (2020). Iq-tree 2: new models and efficient methods for phylogenetic inference in the genomic era. Mol. Biol. Evol. 37, 1530–1534. 10.1093/molbev/msaa015 32011700 PMC7182206

[B17] Nelesen S. Liu K. Wang L.-S. Linder C. R. Warnow T. (2012). Dactal: divide-and-conquer trees (almost) without alignments. Bioinformatics 28, i274–i282. 10.1093/bioinformatics/bts218 22689772 PMC3371850

[B18] Ng A. Jordan M. Weiss Y. (2001). On spectral clustering: analysis and an algorithm. Adv. neural Inf. Process. Syst. 14.

[B19] Page R. D. (2002). “Modified mincut supertrees,” in International workshop on algorithms in bioinformatics (Springer), 537–551.

[B20] Pedregosa F. Varoquaux G. Gramfort A. Michel V. Thirion B. Grisel O. (2011). Scikit-learn: machine learning in Python. J. Mach. Learn. Res. 12, 2825–2830.

[B21] Picard J.-C. Queyranne M. (1980). On the structure of all minimum cuts in a network and applications. Springer.

[B22] Ragan M. A. (1992). Phylogenetic inference based on matrix representation of trees. Mol. phylogenetics Evol. 1, 53–58. 10.1016/1055-7903(92)90035-f 1342924

[B23] Ranwez V. Criscuolo A. Douzery E. J. (2010). Supertriplets: a triplet-based supertree approach to phylogenomics. Bioinformatics 26, i115–i123. 10.1093/bioinformatics/btq196 20529895 PMC2881381

[B24] Robinson D. F. Foulds L. R. (1981). Comparison of phylogenetic trees. Math. Biosci. 53, 131–147. 10.1016/0025-5564(81)90043-2

[B25] Roshan U. W. Moret B. M. Warnow T. Williams T. L. (2003). *Greedy strict-consensus merger: a new method to combine multiple phylogenetic trees* (Citeseer)

[B26] Roshan U. W. Warnow T. Moret B. M. Williams T. L. (2004). “Rec-i-dcm3: a fast algorithmic technique for reconstructing phylogenetic trees,” in Proceedings. 2004 IEEE computational systems bioinformatics conference, 2004 (IEEE), 98–109. 10.1109/csb.2004.1332422 16448004

[B27] Semple C. Steel M. (2000). A supertree method for rooted trees. Discrete Appl. Math. 105, 147–158. 10.1016/s0166-218x(00)00202-x

[B28] Shi J. Malik J. (2000). Normalized cuts and image segmentation. IEEE Trans. pattern analysis Mach. Intell. 22, 888–905. 10.1109/34.868688

[B29] Smith M. R. (2020). Information theoretic generalized robinson–foulds metrics for comparing phylogenetic trees. Bioinformatics 36, 5007–5013. 10.1093/bioinformatics/btaa614 32619004

[B30] Stamatakis A. (2006). Raxml-vi-hpc: maximum likelihood-based phylogenetic analyses with thousands of taxa and mixed models. Bioinformatics 22, 2688–2690. 10.1093/bioinformatics/btl446 16928733

[B31] Steel M. Bocker S. (2000). Simple but fundamental limitations on supertree and consensus tree methods. Syst. Biol. 49, 363–368. 10.1093/sysbio/49.2.363 12118411

[B32] Sumner J. Fernández-Sánchez J. Jarvis P. (2012). Lie markov models. J. Theor. Biol. 298, 16–31. 10.1016/j.jtbi.2011.12.017 22212913

[B33] Swenson M. S. Barbançon F. Warnow T. Linder C. R. (2010). A simulation study comparing supertree and combined analysis methods using smidgen. Algorithms Mol. Biol. 5, 8–16. 10.1186/1748-7188-5-8 20047664 PMC2837663

[B34] Swenson M. S. Suri R. Linder C. R. Warnow T. (2012). Superfine: fast and accurate supertree estimation. Syst. Biol. 61, 214–227. 10.1093/sysbio/syr092 21934137

[B35] Vachaspati P. Warnow T. (2017). Fastrfs: fast and accurate robinson-foulds supertrees using constrained exact optimization. Bioinformatics 33, 631–639. 10.1093/bioinformatics/btw600 27663499 PMC5870905

[B36] Von Luxburg U. (2007). A tutorial on spectral clustering. Statistics Comput. 17, 395–416. 10.1007/s11222-007-9033-z

[B37] Yap V. B. Speed T. (2005). Rooting a phylogenetic tree with nonreversible substitution models. BMC Evol. Biol. 5 (2), 2. 10.1186/1471-2148-5-2 15629063 PMC544347

[B38] Yoshida R. Fukumizu K. Vogiatzis C. (2019). Multilocus phylogenetic analysis with gene tree clustering. Ann. Operations Res. 276, 293–313. 10.1007/s10479-017-2456-9

