# Supplementary material for: Spectral cluster supertree: fast and statistically robust merging of rooted phylogenetic trees
Source: Front Mol Biosci. 2024 Oct 30;11:1432495. doi: 10.3389/fmolb.2024.1432495 (PMC11561713; doi:10.3389/fmolb.2024.1432495)
Supplement: Supplementary file 1 [file DataSheet1.PDF]

Supplementary Material

1 OVERVIEW

The supplementary material contains figures displaying the full results of Spectral Cluster Supertree (SCS) against Bad Clade Deletion (BCD) Fleischauer and Böcker (2017) over all datasets. Unlike the paper, it also includes the F_1 score as defined there in each of the figures. An example calculation is shown in Figure S1. It is notable however that there is a direct mapping between the F_1 score and the Robinson-Foulds Robinson and Foulds (1981) distance, as illustrated by the main paper. For the figures in this supplementary material, higher values are better only for the F_1 score. Lower values are better for all other graphs. Time results are shown on a logarithmic scale. The code used to generate these figures, as well as the raw results, has been archived online (https://doi.org/10.5281/zenodo.11118313). We divide the supplementary material into two parts. The first are extra figures that are referenced to by the paper that do not include the F_1 score for better readability. The second part shows all figures that include the F_1 score for completeness.

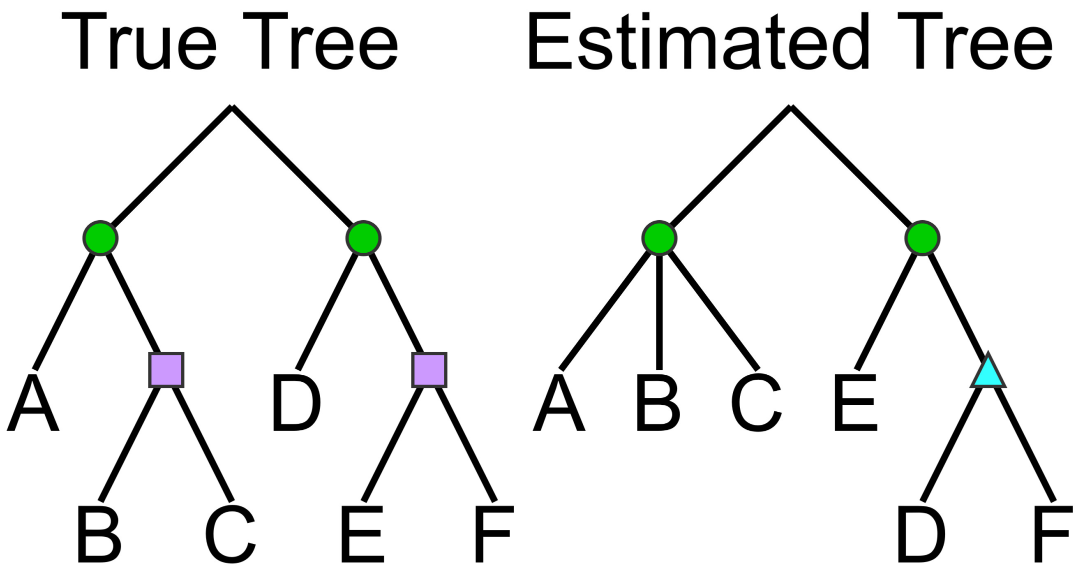

Figure S1. An example of how to calculate the rooted variant of the F_1 score. True positive clades are those present both in the true and estimated trees (marked by green circles); there are two such clades here. False positive clades are those present in the estimated tree, but not the true tree (marked by single cyan triangle). False negative clades appear in the true tree but not in the estimated tree (marked by the two purple squares). $F_1 = \frac{2TP}{2TP + FP + FN} = \frac{4}{4+2+1} \approx 0.57$

2 EXTRA FIGURES WITHOUT F_1 SCORE

2.1 SCS-DCM-IQ Dataset

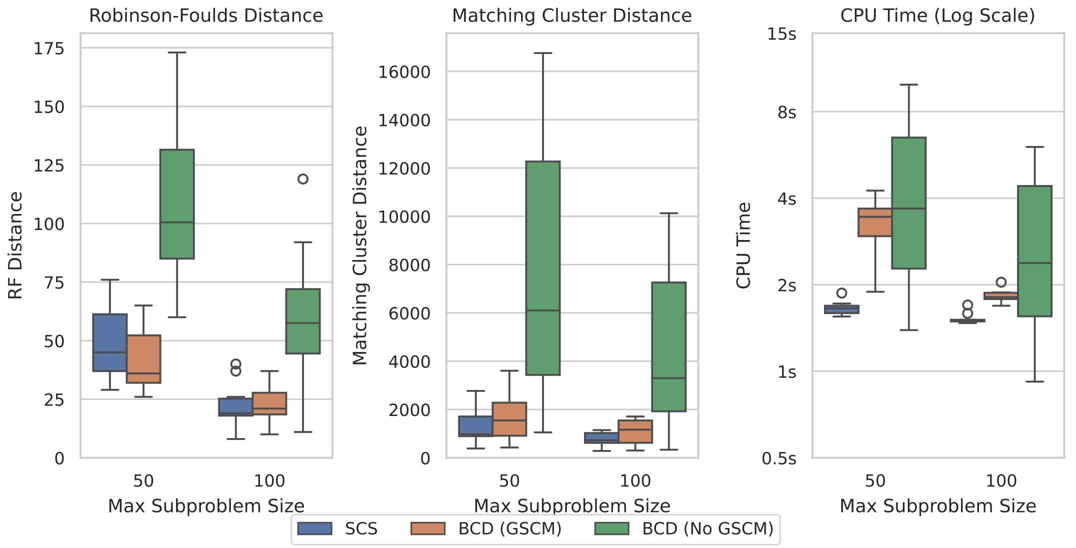

Figure S2. Spectral Cluster Supertree vs Bad Clade Deletion on the SCS DCM IQ-TREE dataset with 500 taxa. Each parameterisation contains 10 problems.

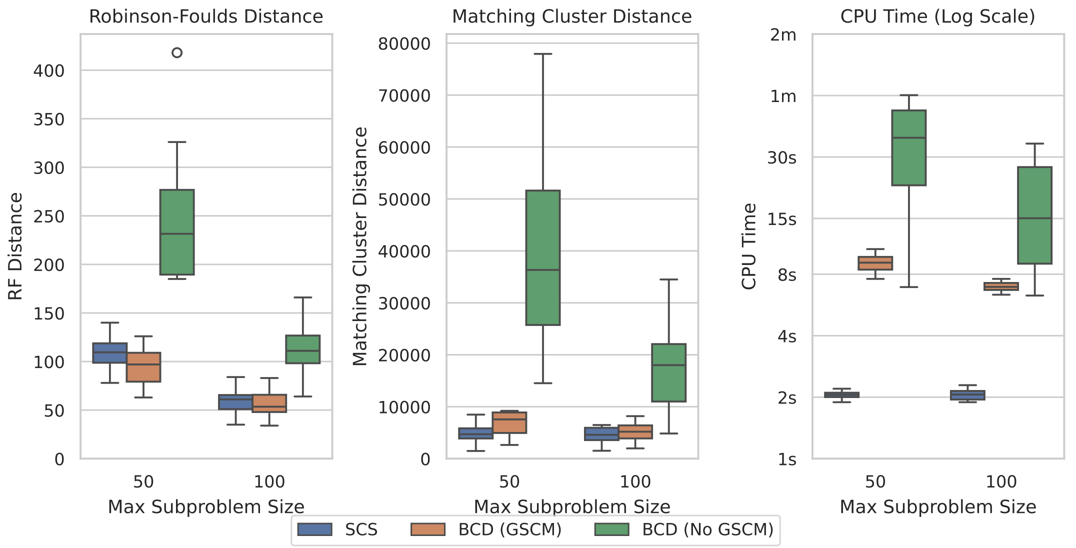

Figure S3. Spectral Cluster Supertree vs Bad Clade Deletion on the SCS DCM IQ-TREE dataset with 1000 taxa. Each parameterisation contains 10 problems.

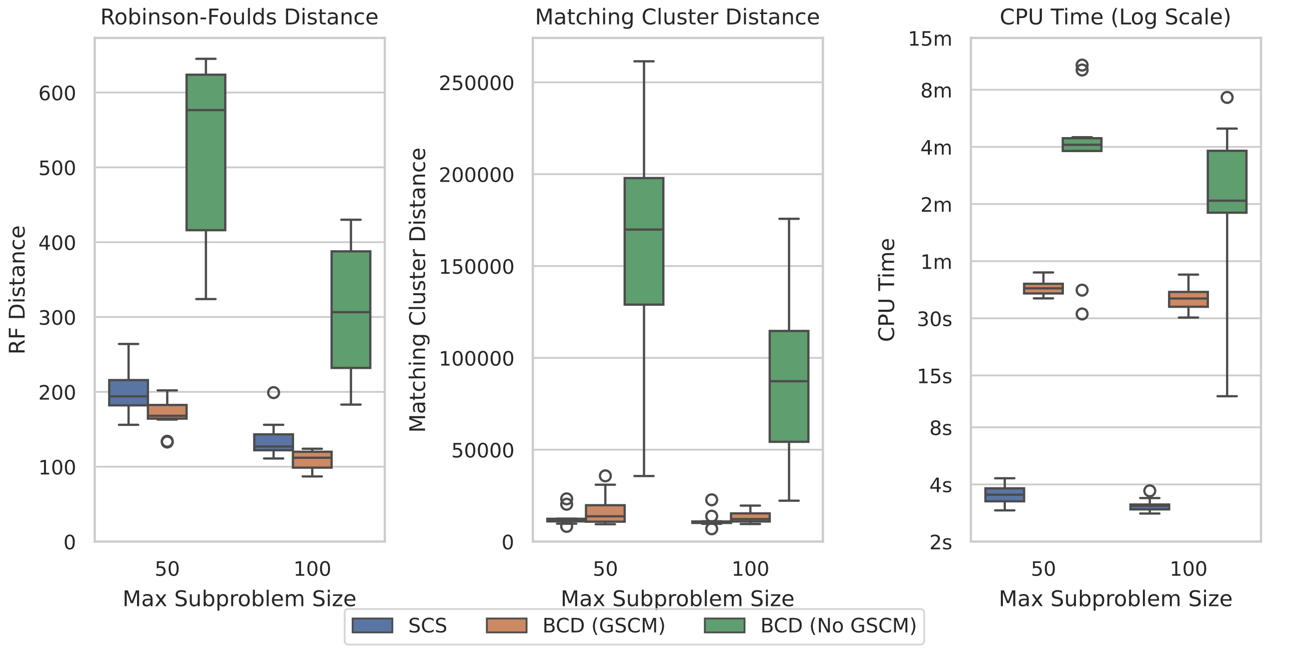

Figure S4. Spectral Cluster Supertree vs Bad Clade Deletion on the SCS DCM IQ-TREE dataset with 2000 taxa. Each parameterisation contains 10 problems.

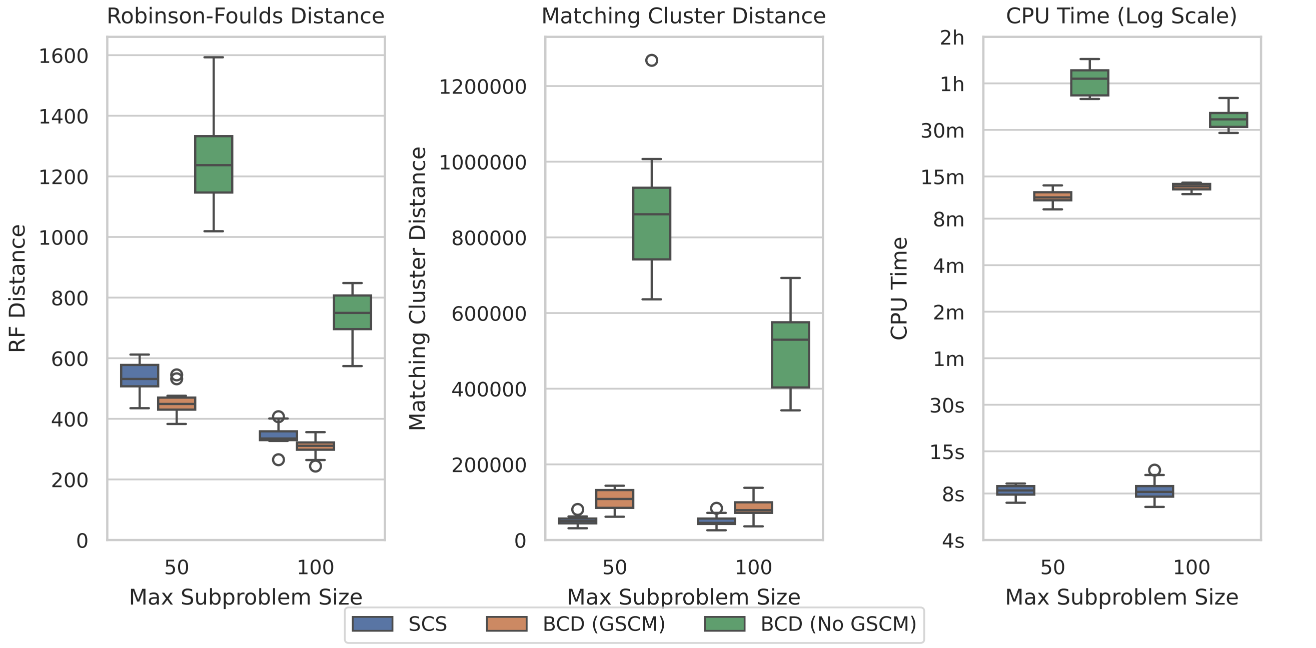

Figure S5. Spectral Cluster Supertree vs Bad Clade Deletion on the SCS DCM IQ-TREE dataset with 5000 taxa. Each parameterisation contains 10 problems.

2.2 SMIDGenOG Dataset

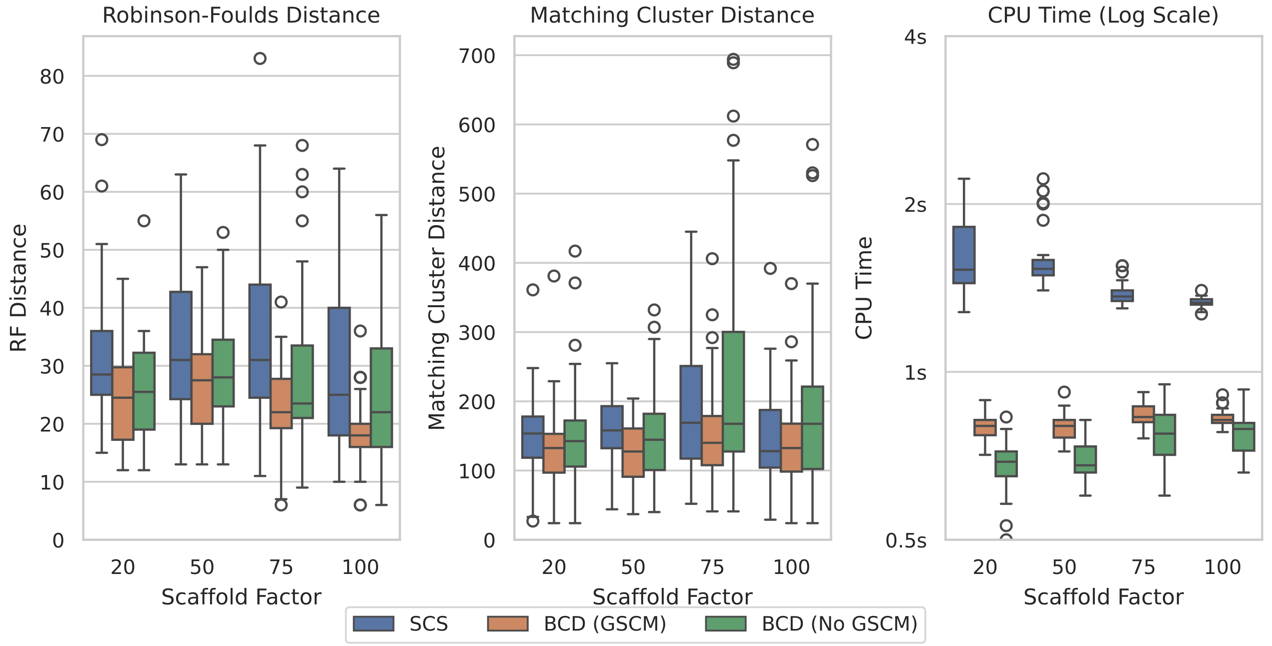

Figure S6. Spectral Cluster Supertree vs Bad Clade Deletion on the SMIDGenOG dataset with 100 taxa. Each parameterisation contains 30 problems.

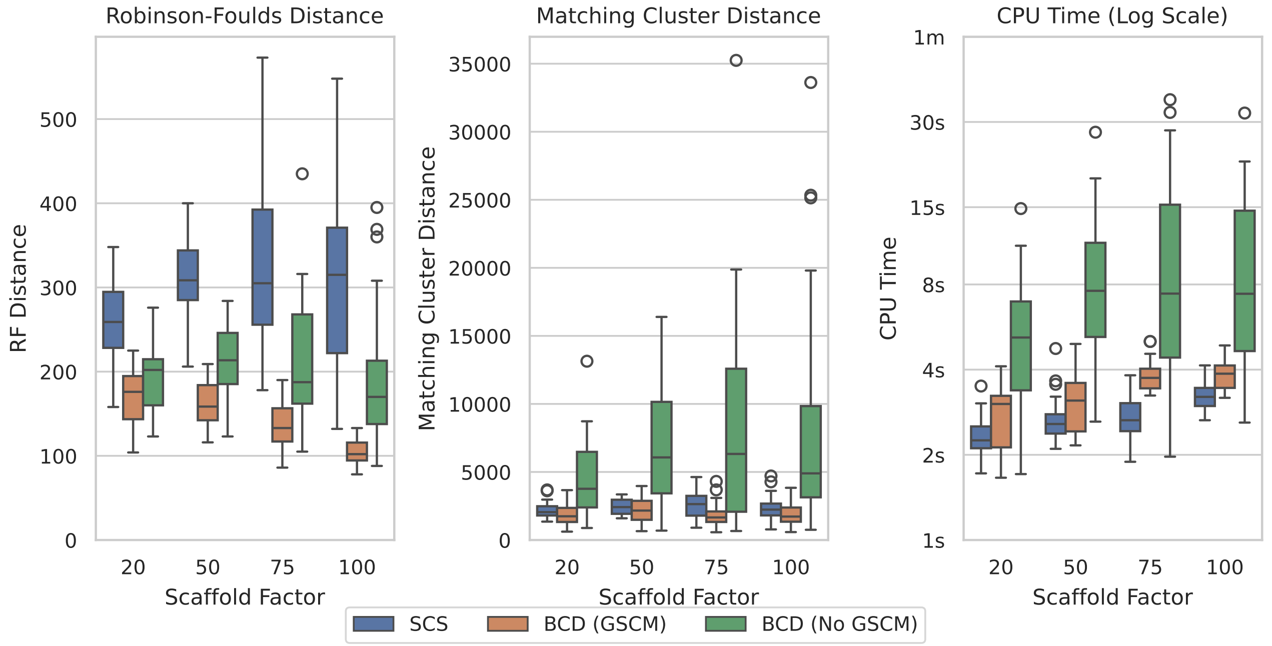

Figure S7. Spectral Cluster Supertree vs Bad Clade Deletion on the SMIDGenOG dataset with 500 taxa. Each parameterisation contains 30 problems.

2.3 SuperTriplets Dataset

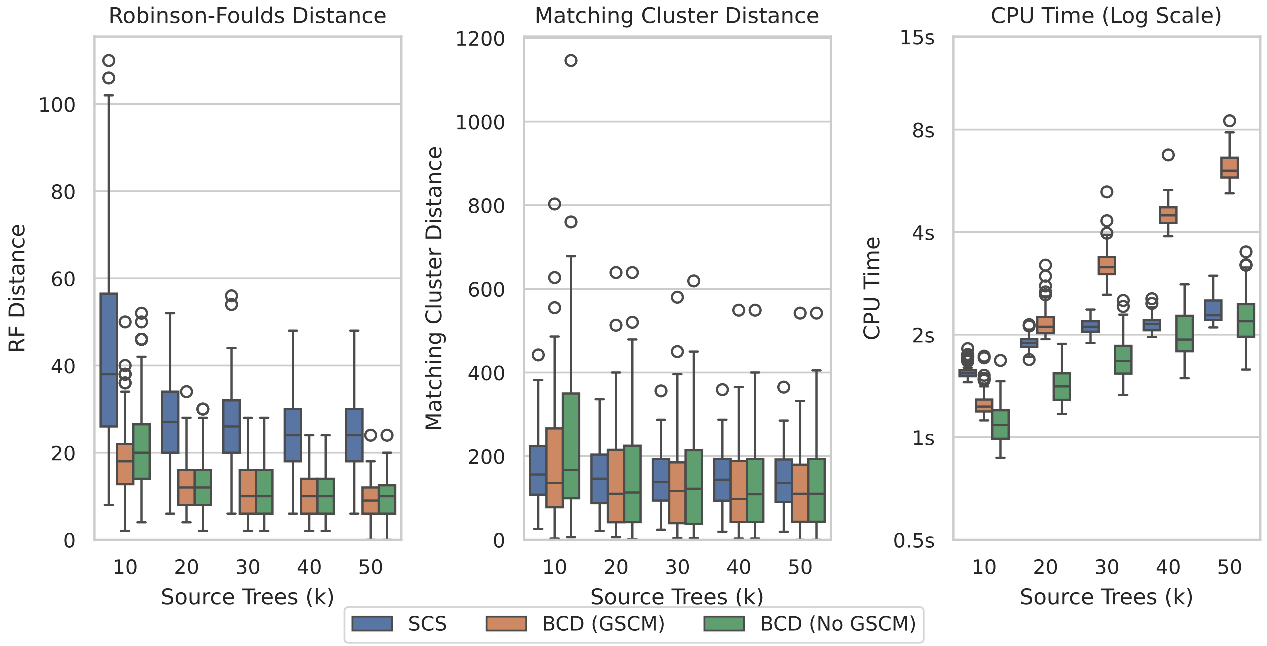

Figure S8. Spectral Cluster Supertree vs Bad Clade Deletion on the SuperTriplets dataset with a deletion rate of 25%. Each parameterisation contains 100 problems.

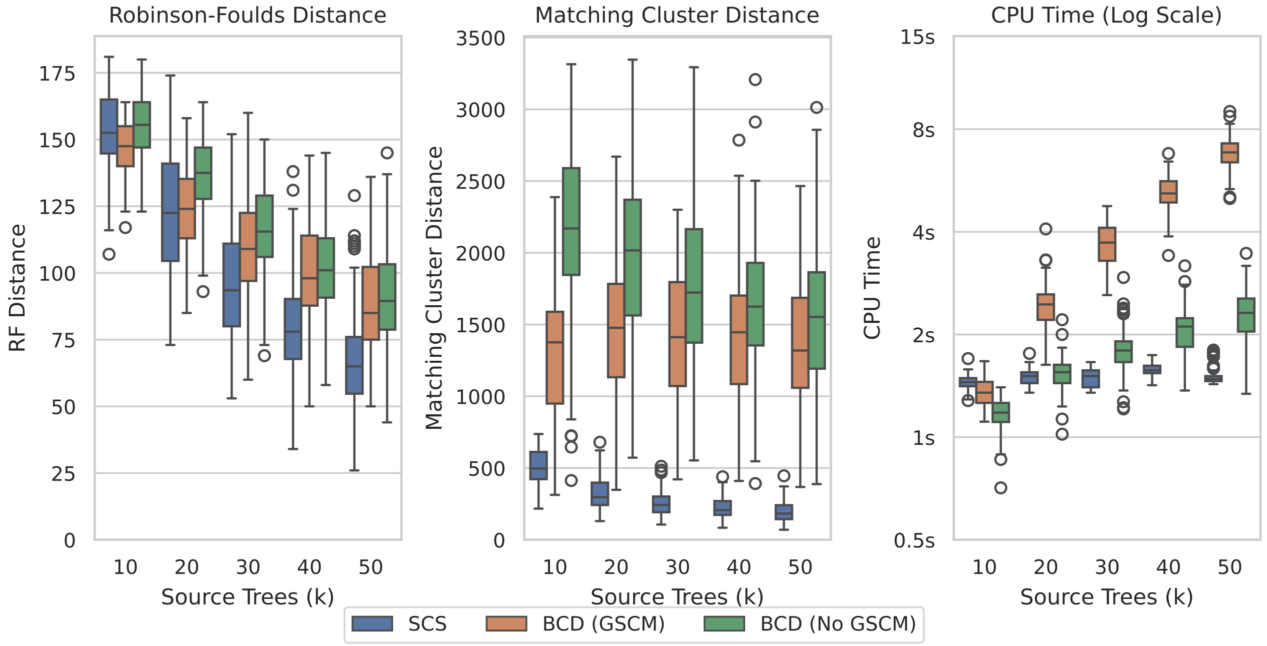

Figure S9. Spectral Cluster Supertree vs Bad Clade Deletion on the SuperTriplets dataset with a deletion rate of 75%. Each parameterisation contains 100 problems.

3 FIGURES WITH F_1 SCORE

3.1 SCS-DCM-IQ Dataset

Our SCS-DCM-IQ dataset was created to mimic what may be encountered by divide and conquer algorithms for phylogenetic reconstruction. Figures S10-S14 compare SCS to BCD with increasing amounts of taxa. While BCD (GSCM) tends to achieve more exactly correct clades (RF distance Robinson and Foulds (1981) and F_1 score Fleischauer and Böcker (2017)), the overall topological accuracy of SCS appears to be consistently superior (Matching Cluster distance Bogdanowicz and Giaro (2013)). SCS is also much faster than BCD, on the largest dataset taking on average ~20 seconds per problem, where BCD takes ~2 hours.

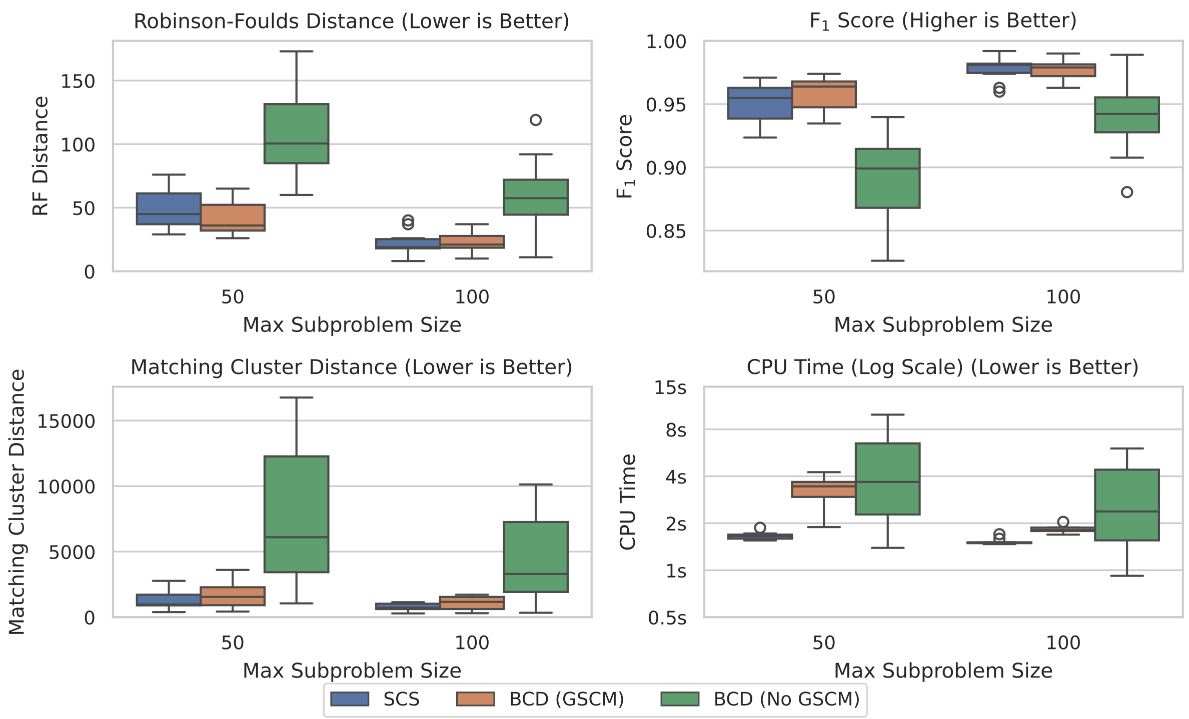

Figure S10. SCS vs BCD on the SCS-DCM-IQ dataset with 500 taxa. All methods solved all problems within the timeout.

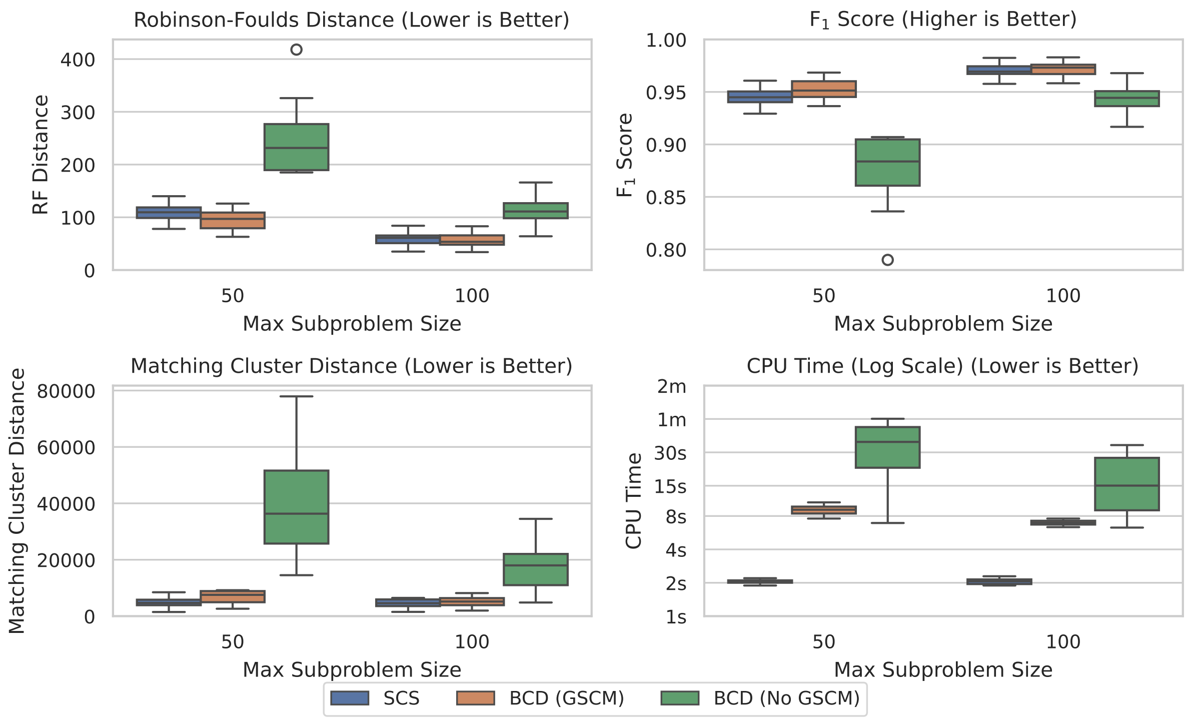

Figure S11. SCS vs BCD on the SCS-DCM-IQ dataset with 1000 taxa. All methods solved all problems within the timeout.

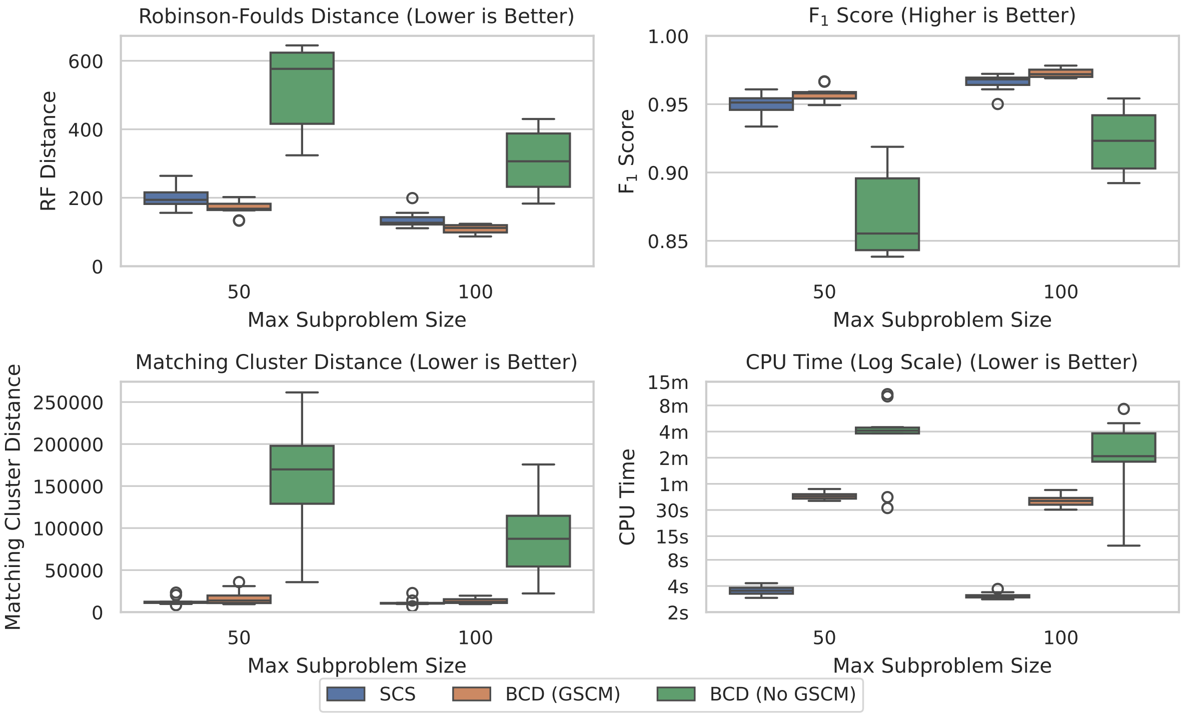

Figure S12. SCS vs BCD on the SCS-DCM-IQ dataset with 2000 taxa. All methods solved all problems within the timeout.

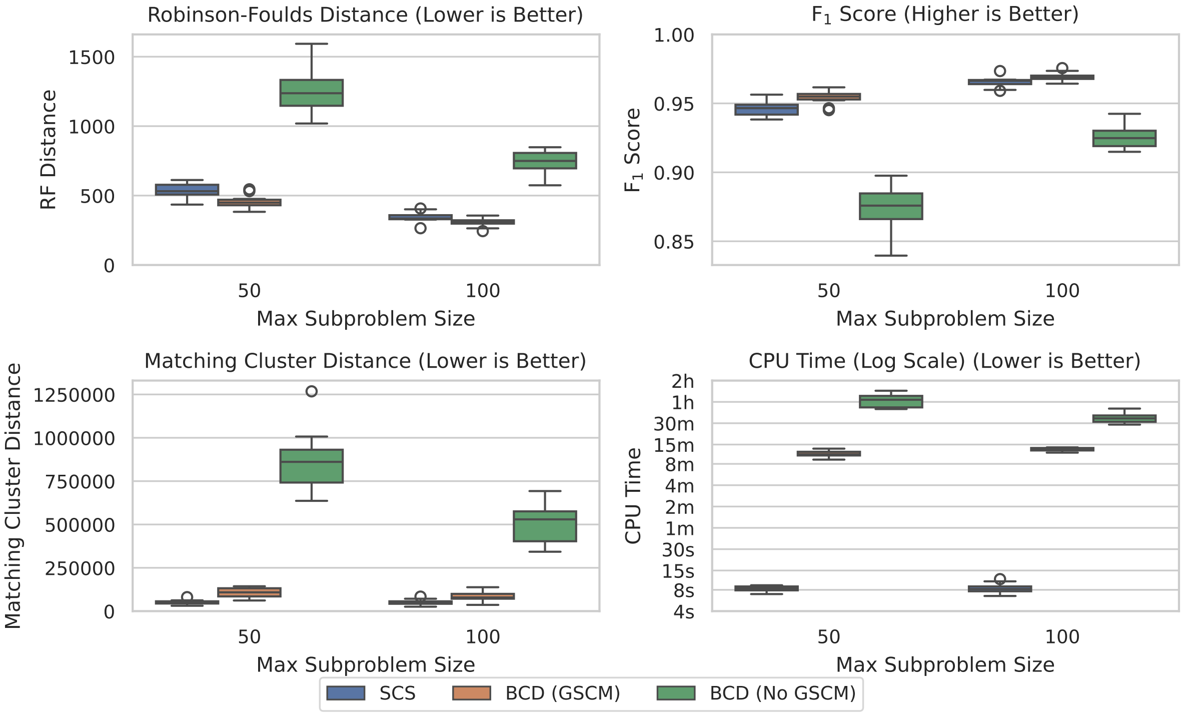

Figure S13. SCS vs BCD on the SCS-DCM-IQ dataset with 5000 taxa. All methods solved all problems within the timeout.

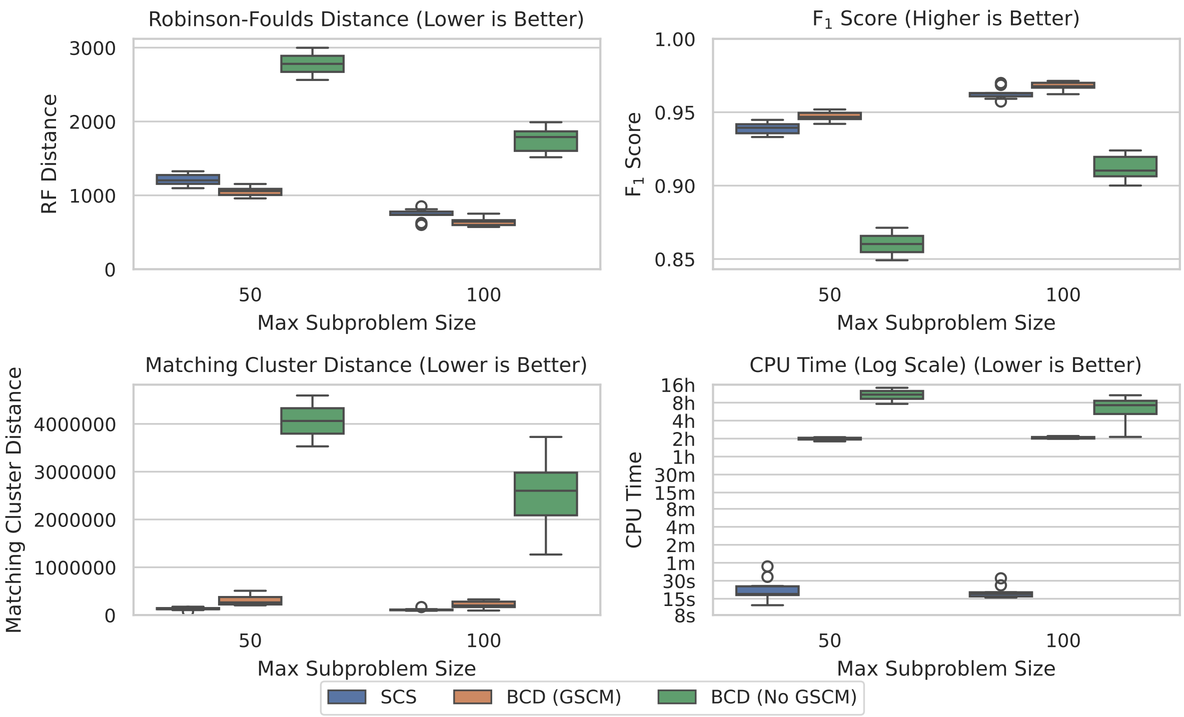

Figure S14. SCS vs BCD on the SCS-DCM-IQ dataset with 10000 taxa. BCD without GSCM processing only solved the first 2 and 6 problems within the timeout for the 50 and 100 max subproblem sizes respectively. The other methods solved all ten problems.

3.2 SMIDGenOG-5500 Dataset

The SMIDGenOG-5500 dataset Fleischauer and Böcker (2017) is a large scale dataset containing on average 5500 taxa. It follows the SMIDGen protocol Swenson et al. (2010), differing in how it generates scaffold trees due to the scale of the dataset. It aims to emulate what may be encountered by systematists at a large scale. Figure S15 compares SCS to BCD over this dataset. While BCD (GSCM) again achieves more exactly correct clades, the overall topological accuracy of the tree for SCS is vastly superior. SCS takes on the order of a couple of minutes to solve these problems, whereas BCD can take multiple hours.

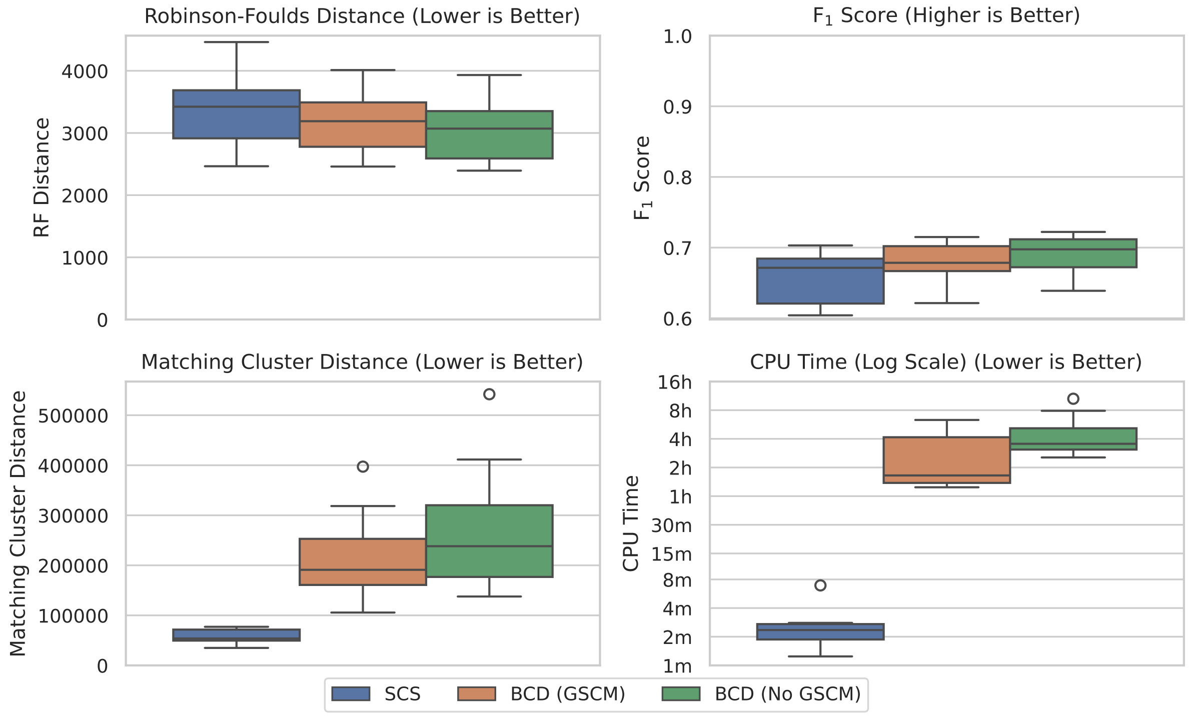

Figure S15. SCS vs BCD on the SMIDGenOG-5500 dataset. As there is no parameterisation for this dataset, no x-axis is displayed. All methods solved all problem instances within the timeout.

3.3 SMIDGenOG Dataset

The SMIDGenOG dataset Fleischauer and Böcker (2016) was developed using the SMIDGen protocol Swenson et al. (2010) in a rooted context. The datasets generated through the SMIDGen protocol aims to imitate data collection processes typically used by systematists. The dataset contains a scaffold tree sampling a "Scaffold Factor" percentage of the taxa, and many densely sampled clade based source trees. Figures S16-S18 compares SCS to BCD in increasing order of number of taxa. BCD (GSCM) appears to perform better than SCS over this dataset on all accuracy metrics, though the degree of improvement appears to decrease as the scaffold factor decreases. The time results are all small enough to not matter for practical purposes between SCS and BCD (GSCM).

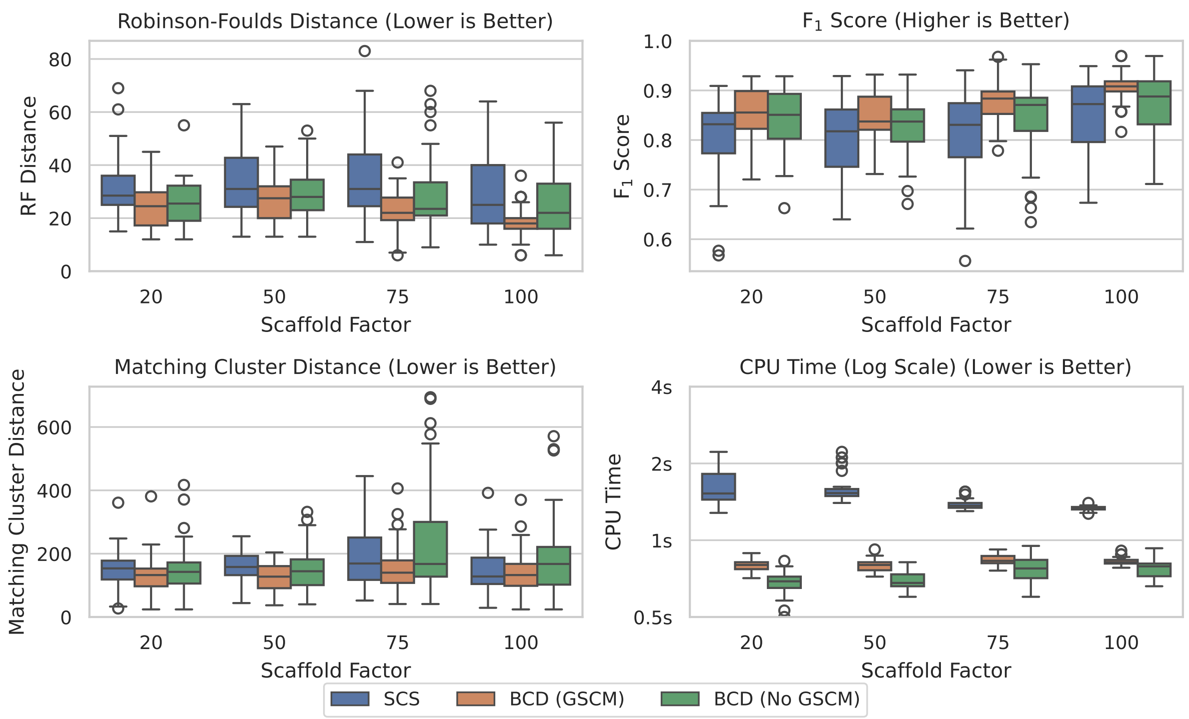

Figure S16. SCS vs BCD on the SMIDGenOG dataset with 100 taxa. The SMIDGenOG dataset contains one widely sampled scaffold tree, and many densely sampled clade-based source trees.

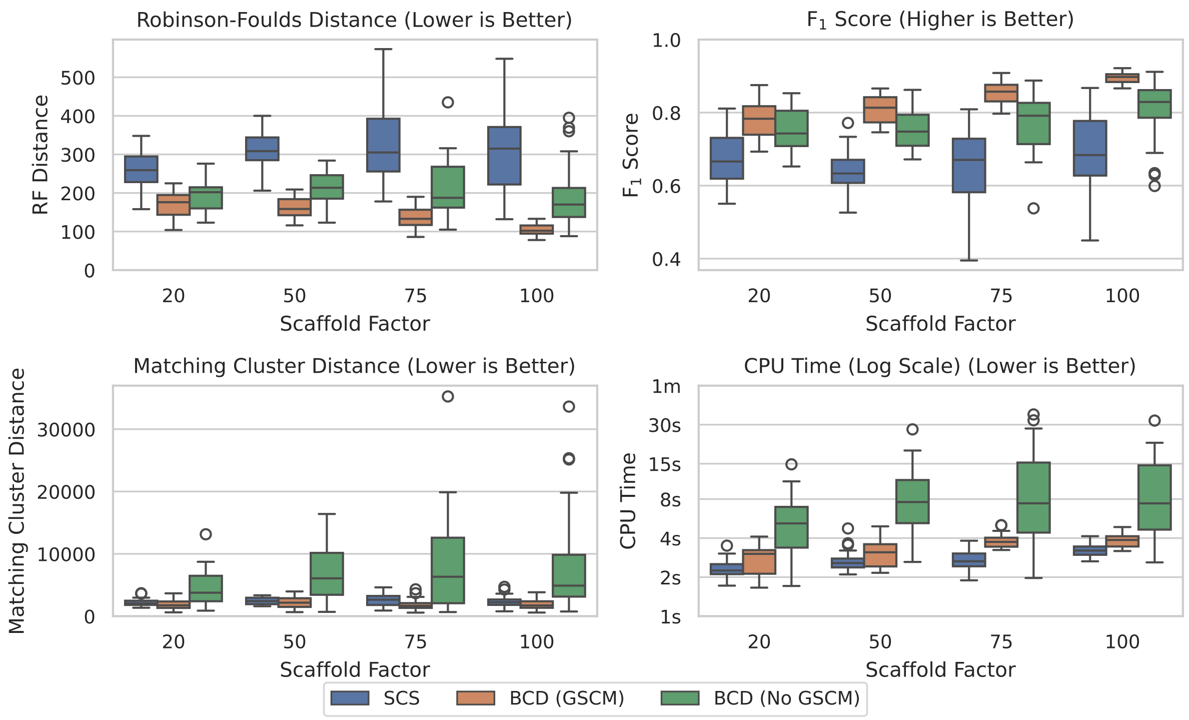

Figure S17. SCS vs BCD on the SMIDGenOG dataset with 500 taxa. The SMIDGenOG dataset contains one widely sampled scaffold tree, and many densely sampled clade-based source trees.

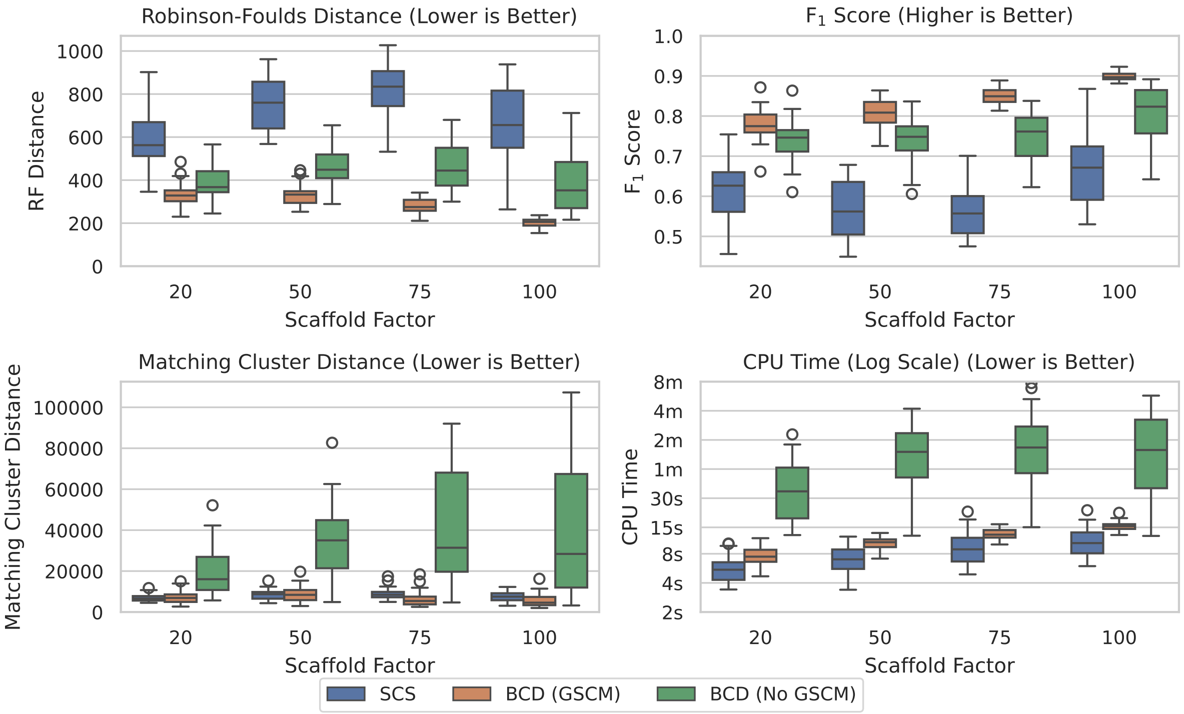

Figure S18. SCS vs BCD on the SMIDGenOG dataset with 1000 taxa. The SMIDGenOG dataset contains one widely sampled scaffold tree, and many densely sampled clade-based source trees.

3.4 SuperTriplets Dataset

The SuperTriplets dataset Ranwez et al. (2010) explores the effect of the number of taxa present in each of the source trees after a percentage of them are removed, and the number of source trees, on supertree reconstruction accuracy. Figures S19-S21 compare BCD to SCS in increasing order of deletion rate. For the 25% deletion rate, SCS performs worse in terms of accuracy for the RF and F_1 metrics, though has a lower spread of values (albeit higher median) for the Matching Cluster distance. As the deletion rate of taxa increases (i.e. the number of taxa represented in each source tree decreases), the degree of improvement of SCS over BCD increases with respect to the Matching Cluster distance in particular. SCS even generally outperforms BCD in terms of the RF distance for the 75% deletion rate. The time results here are all low enough to not matter for practical purposes.

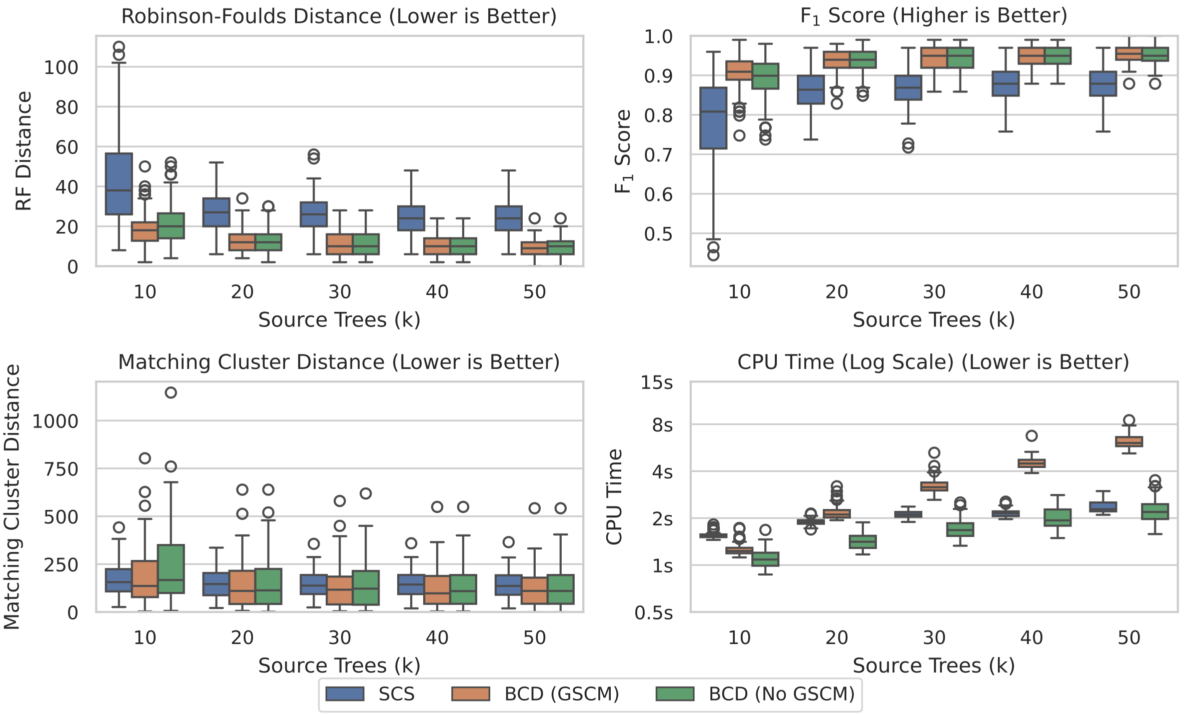

Figure S19. SCS vs BCD on the SuperTriplets dataset with a deletion rate of 25%.

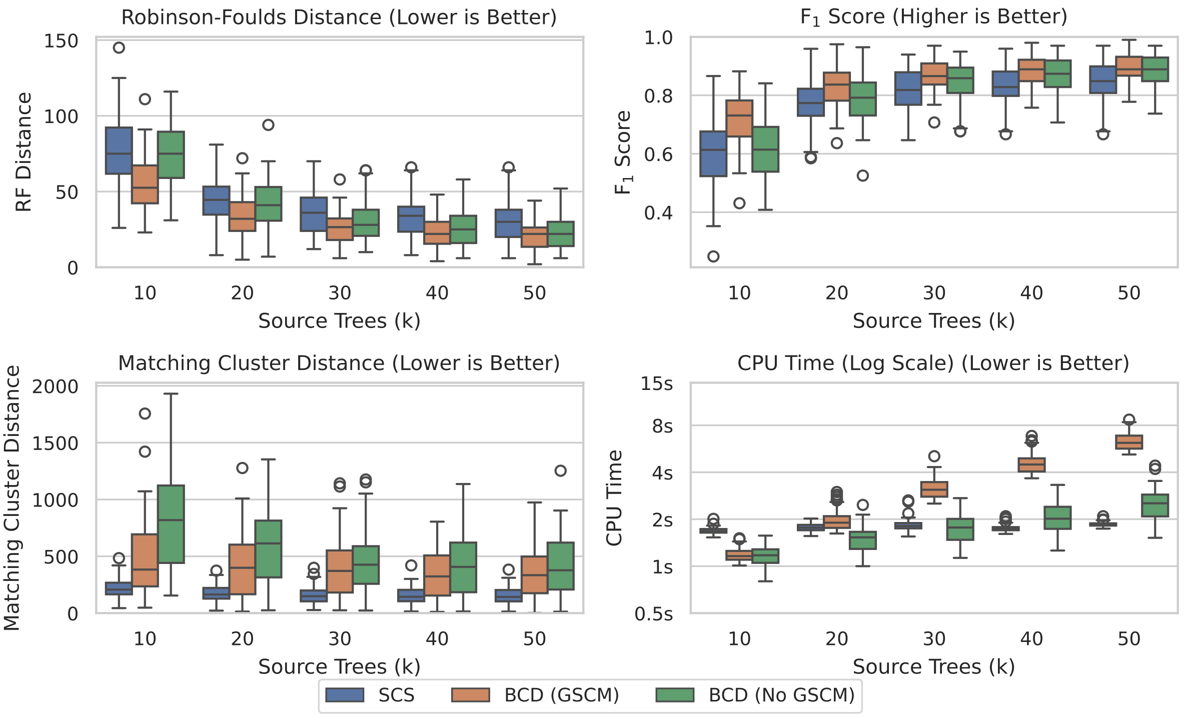

Figure S20. SCS vs BCD on the SuperTriplets dataset with a deletion rate of 50%.

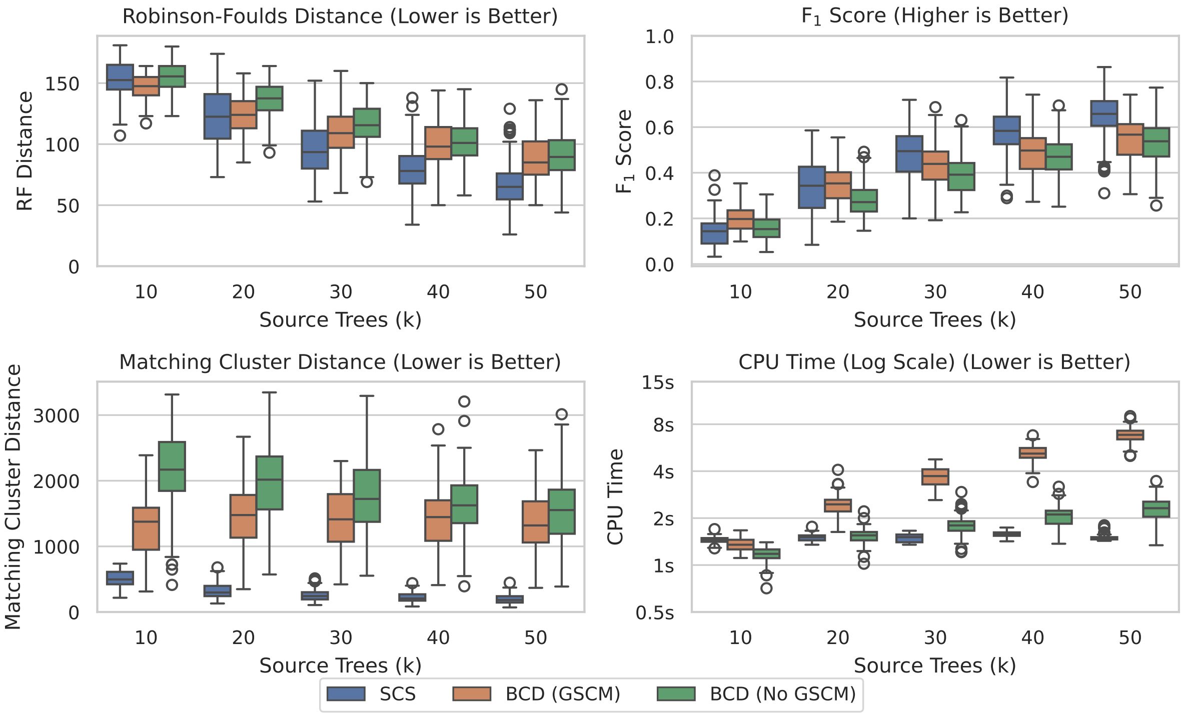

Figure S21. SCS vs BCD on the SuperTriplets dataset with a deletion rate of 75%.

REFERENCES

Bogdanowicz, D. and Giaro, K. (2013). On a matching distance between rooted phylogenetic trees. *International Journal of Applied Mathematics and Computer Science* 23, 669–684

Fleischauer, M. and Böcker, S. (2016). Collecting reliable clades using the greedy strict consensus merger. *PeerJ* 4, e2172

Fleischauer, M. and Böcker, S. (2017). Bad clade deletion supertrees: a fast and accurate supertree algorithm. *Molecular biology and evolution* 34, 2408–2421

Ranwez, V., Criscuolo, A., and Douzery, E. J. (2010). Supertriplets: a triplet-based supertree approach to phylogenomics. *Bioinformatics* 26, i115–i123

Robinson, D. F. and Foulds, L. R. (1981). Comparison of phylogenetic trees. *Mathematical biosciences* 53, 131–147

Swenson, M. S., Barbançon, F., Warnow, T., and Linder, C. R. (2010). A simulation study comparing supertree and combined analysis methods using smidgen. *Algorithms for Molecular Biology* 5, 1–16
